# Functional Impairment in Individuals Exposed to Violence Based on Electronical Forensic Medical Record Mining and Their Profile Identification: Controlled Observational Study

**DOI:** 10.2196/43563

**Published:** 2024-09-27

**Authors:** Ivan Lerner, Patrick Chariot, Thomas Lefèvre

**Affiliations:** 1 Department of Legal and Social Medicine Hôpital Jean-Verdier, Assistance publique - Hôpitaux de Paris Bondy France; 2 Unité mixte de recherche 8156 Centre national pour la recherche scientifique – Unité 997 Institut national de la santé et de la recherche médicale – Ecole des hautes études en sciences sociales – Université Sorbonne Paris Nord Institut de Recherche Interdisciplinaire Sur les Enjeux Sociaux Aubervilliers France

**Keywords:** reproducibility, interpersonal violence, functional impairment, psychological trauma, clustering, intimate partner violence

## Abstract

**Background:**

Little is known about the functional consequences of violence when directly assessed as a primary outcome, and even less about how consistently these consequences are evaluated in a judicial context. The World Health Organization (WHO) highlighted the importance of a functional approach to health in 2001 with the release of the International Classification of Functioning, Disability, and Health (ICF). In most European countries, forensic physicians assess individuals exposed to violence to evaluate the outcomes of violence, providing certified medical evidence for magistrates’ sentencing decisions. This evaluation involves a mix of objective, subjective, and contextual elements, such as reported symptoms of fear, pain, and details of the assault. Quantifying these subjective elements with scales could enhance their interpretation and application in a judicial context.

**Objective:**

This study aims to (1) characterize and (2) assess 6 scales measuring subjective elements of functional impairment among individuals exposed to violence.

**Methods:**

We conducted a retrospective study that included individuals exposed to violence examined in a French department of forensic medicine over 12 months. A typology of violence encountered in medical settings was built based on the mining of electronic health records and the use of pattern recognition algorithms. The optimal number of violence types was determined using a robust and stable clustering approach, involving sample resampling and a multimetric scheme. Patients were then paired according to their homogeneous profiles, and the intra- and interrater reproducibility of the scales was evaluated.

**Results:**

All pain, fear, and life threat scales were significantly associated with higher functional impairment, suggesting that these measures contribute to the overall assessment of functional impairment. The intra- and interrater reproducibility of scales among similar situations of violence was measured, ranging from mild to good, with coefficients of concordance between 0.46-0.66 and 0.43-0.66, respectively. Individuals reporting intimate partner violence showed higher scores in both fear and perception of a life threat during the assault and medical interview, while individuals reporting battery by multiple unknown assailants presented higher scores only in perception of a life threat during the assault. We identified 5 remarkably stable profiles of situations of violence, consistent with clinical practice.

**Conclusions:**

Pain, fear, and life threat scales were related to functional impairment according to expert knowledge and demonstrated fair reproducibility under real-life conditions for similar situations of violence. Subjective elements related to functional impairment in individuals exposed to violence can be quantified using Likert scales during medical interviews.

## Introduction

Many individuals experience both intended and unintended acts of violence, leading to physical and psychological trauma that impacts their everyday functioning [1]. Intimate partner homicides constitute 1 in 7 homicide cases and account for 1 in 3 female homicides [2]. In the United States, studies have reported that intimate partner violence (IPV) with health, legal, or work-related impacts affects 28.8% of women and 9.9% of men over their lifetime [3]. Additionally, 76%-82% of young adults experience community violence during their lifetime [4]. Evaluating functional impairment among individuals exposed to violence has significant implications for patient care and the judicial system. While the World Health Organization (WHO) and many countries worldwide recognized the functional approach to pathology and health through the adoption of the International Classification of Functioning, Disability, and Health (ICF) in 2001, little to no research has been conducted on functional impairment in individuals exposed to violence. In most European countries, forensic physicians examine individuals exposed to violence to assess the outcomes of violence at the request of magistrates, who can base their sentences on certified medical evidence [5]. To evaluate the functional impairment following an assault, physicians rely on both objective, observable elements and subjective elements, such as patient-reported symptoms (eg, expressions of fear or pain). Legal authorities may underestimate the importance of subjective elements compared with objective ones [6]. Indeed, such elements are not systematically reported in medical documents by physicians, or they may lack standardized, consensual measures of these elements. Additionally, psychological trauma is primarily assessed based on patients reporting subjective symptoms. As a result, sources of psychological trauma leading to functional impairment may be considered less than physical ones because they are perceived as less reliable in a judicial context compared with physical, observable symptoms [6]. However, psychological trauma is a major source of functional impairment among individuals exposed to violence [7] and can potentially lead to long-term health deterioration [8]. A more systematic and comprehensive assessment of psychological trauma would enhance its early detection and facilitate the treatment of severe psychiatric conditions such as posttraumatic stress disorder [9,10].

One solution to address these issues is to use scales that quantify the intensity of subjective elements relevant to assessing functional impairment. Such tools offer several advantages. First, they can simplify the complex analysis of multiple and variably reported symptoms into a cohesive index. Second, they allow for comparisons between different patients or between different measurements at various end points for the same patient. Finally, because it is possible to incorporate both physician-rated and self-rated assessments, these tools should provide information that more accurately reflects the response of individuals exposed to violence rather than solely the physician’s perspective.

Such scales have been developed in other contexts to measure elements such as pain [11] or daily instrumental activities [12], in both research settings and everyday clinical practice. However, these scales may not meet the expectations for examining individuals exposed to violence, as they can be too time-consuming or are not validated for this particular clinical setting. To be effective in everyday practice, scales should meet 4 criteria: (1) they should consist of brief, straightforward questions that are easily understood by patients during routine assessments; (2) they should contribute to the overall evaluation of functional impairment; (3) they should exhibit sufficient concordance, demonstrating intrarater reproducibility (consistency when the same physician asks the same questions) and interrater reproducibility (consistency between different physicians asking the same questions) in comparable situations involving violence; and (4) they should be easily comprehensible and allow for straightforward interpretation by legal authorities, such as judges or judicial police officers.

This study aimed to investigate, under real-life conditions, the contribution of 6 scales to the global assessment of functional impairment and their concordance. These scales were based on a comprehensive, real-life typology of violence. Three self-rated scales measured subjective elements: perceptions of pain, fear, and life threat. These subjective elements were measured at 2 distinct end points: at the time of the assault and at the time of medical consultation. Two physician-rated scales assessed the intensity of functional impairment and the quality of interaction between the patient and the physician during the consultation. Finally, the psychosomatic index evaluated the relative importance of psychological trauma in functional impairment.

The primary objective of this study was to characterize scales measuring subjective elements related to functional impairment in individuals exposed to violence within a judicial context. The evaluation of the scales’ reproducibility was based on a typology of situations involving violence, determined through the analysis of extensive multivariable observational data contained in electronic health records. We used this typology to control for potential confusion biases and variability in real-life practice settings, providing a comprehensive assessment of the relationships between practitioners and scale results. The secondary objective was to describe the degree of functional impairment and psychological trauma–related symptoms across this typology of situations involving violence.

## Methods

### Overview

In this retrospective study, data were extracted for all consecutive individuals exposed to violence examined by physicians in the Department of Forensic Medicine at Jean-Verdier Hospital in Bondy (Seine-Saint-Denis, France), in the Paris metropolitan area, between January 1, 2015, and December 31, 2015. These data are not publicly available due to privacy restrictions, as they contain information that could compromise the confidentiality of research participants. The data used in this study resulted from the combination of 2 types of sources. On the one hand, it included systematically collected, routine, and standardized characteristics such as age, sex, and circumstances of violence (eg, locations). On the other hand, it involved retrieving corresponding medical certificates from electronic health records and extracting additional characteristics not included in the standard collection through simple textual analysis techniques (eg, searching for terms, variants, and lexical fields while accounting for typing errors or spelling variations). These analyses are facilitated by the fact that the certificates are in digital form and standardized. The features extracted in this manner primarily concern psychological symptoms.

### Ethics Approval

This research study was conducted retrospectively from data obtained for clinical purposes. An ethics approval of the project (reference number CERHUPO 2015-07-03) was given by the institutional review board (IRB 00001072) CPP Ile-de-France 2 on July 9, 2015. An information note for patients was displayed in each consultation room. This information was also available in the welcome booklet provided to each hospitalized patient. Patients were informed about the potential statistical use of their personal data, which would be anonymized and used solely for research purposes. They could opt out of this use by contacting the department manager.

### Inclusion and Exclusion Criteria

Patients reporting deliberate assault and battery were assessed for eligibility. We excluded patients younger than 10 years, those reporting unintended violence or neglect, those examined more than 30 days after the reported incident, those examined by physicians with fewer than 300 patients per year, and those assessed for a second evaluation of their functional impairment. Only patients with complete data were included in the analysis.

### Description of the Scales

Three self-evaluated Likert scales were used to measure pain, fear, and the perception of a life threat at 2 distinct end points: at the time of the assault and during the medical interview. Each Likert item had 7 levels (from 0=no pain to 6=maximum pain). The physician asked the questions in a general manner and could repeat them up to 2 times while providing a visual graduated scale for the patient to use in self-rating. Regarding the pain scale, the physician asked (translated from French) “On a scale of 0-6, where 0 means no pain and 6 represents the maximum amount of pain you can imagine, how would you rate the pain you experienced during the assault?” followed by, “Similarly, on a scale of 0-6, where 0 means no pain and 6 represents the maximum amount of pain you can imagine, how would you rate the pain you are experiencing now?” Questions about fear and the perception of a life threat were asked in a similar manner (see the “Questions Asked During Consultations” section in Multimedia Appendix 1).

We introduced 2 physician-evaluated Likert scales, each with 7 levels from 0 to 6. One scale was used to measure the intensity of global functional impairment at the time of the consultation, with 0 indicating no functional impairment and 6 representing maximum functional impairment. The other scale was used to assess the quality of interaction between the physician and the patient. Physicians were asked to rate the quality of interaction from 0 to 6, with 0 indicating the worst quality and 6 representing the most satisfying interaction. Finally, we introduced the psychosomatic index, a 7-level scale from 0 to 6. Physicians rated this index from 0 if physical trauma was the only source of functional impairment to 6 if psychological trauma was the only source of functional impairment [13].

### Statistical Analysis

#### Analysis Strategy

We adopted a 2-stage strategy for analysis. First, we developed a typology of situations involving violence based on observational data. We identified homogeneous profiles by examining the most common characteristics among situations of violence, including the characteristics of the individuals exposed to violence, assailants, and the assaults themselves. We hypothesized that within a given type, the characteristics of all situations measured in real-life practice settings are sufficiently homogeneous to allow direct comparisons between practitioners in terms of scale results. Any potential intra- and interrater differences can therefore be attributed to the quality of the scales. Second, we paired patients based on their membership in the same homogeneous profile and evaluated the intrarater and interrater reproducibility of the scales. Details of a sensitivity analysis are provided in the “Sensitivity Analysis” section in Multimedia Appendix 1, and information on the R package (R Foundation for Statistical Computing) used is provided in the “Software Used for Analyses” section in Multimedia Appendix 1 [14-16].

#### Determining a Typology of Situations of Violence

Situations of violence were characterized by the characteristics of individuals exposed to violence and assailants, as well as other circumstances of the assault. Among these, assault outcomes were defined by physical trauma and functional impairment. Functional impairment was quantified, as requested by French judicial authorities, in terms of days of total incapacity to work (TIW) [17,18]. Details are provided in the “Defining Situations Involving Violence” section in Multimedia Appendix 1. We used a clustering algorithm [19] to identify homogeneous profiles of situations involving violence. The Partitioning Around Medoids [20] algorithm was used to automatically detect groups or clusters of similar patients, provided the desired number of groups was specified. We applied the consensus clustering framework [21] to determine the optimal number of clusters. Finally, we investigated the clinical characteristics underlying each violent situation profile.

#### Scales Characterization

First, we described the scales by median and IQR for the overall population and within each violent situation profile. Second, we compared scale scores with related subjective elements. We selected patient-reported symptoms for 5 psychological dimensions related to psychological trauma: sleep disorders (difficulty initiating sleep, frequent awakenings, or early-morning awakenings), loss of appetite, stress symptoms (recurrent memories, avoidance, hypervigilance), expression of pain, and expression of fear. We performed a comparative analysis of scale medians between physicians. We tested for global differences using the Kruskal-Wallis test [22] and for pairwise differences between physicians using the Conover post hoc analysis [23]. Adjustments were made using the Bonferroni method. Finally, we applied univariate linear regression models to determine whether each scale was associated with functional impairment as measured by TIW.

#### Interrater Reproducibility

For each pair of physicians, we randomly matched patients within the same violent situation profile. Thus, for each pair of physicians, we matched patients who experienced similar situations of violence. We used Kendall *W*, or the coefficient of concordance [24,25], to measure reproducibility between raters for the same violent situation profiles. Kendall *W* was corrected for ties (see formula in the “Kendall W Coefficient of Concordance” section of Multimedia Appendix 1).

#### Intrarater Reproducibility

For each physician, we randomly matched patients within the same violent situation profile and used Kendall *W* to measure the reproducibility of scales when a physician examined patients with the same violent situation profile.

## Results

**Figure 1:**
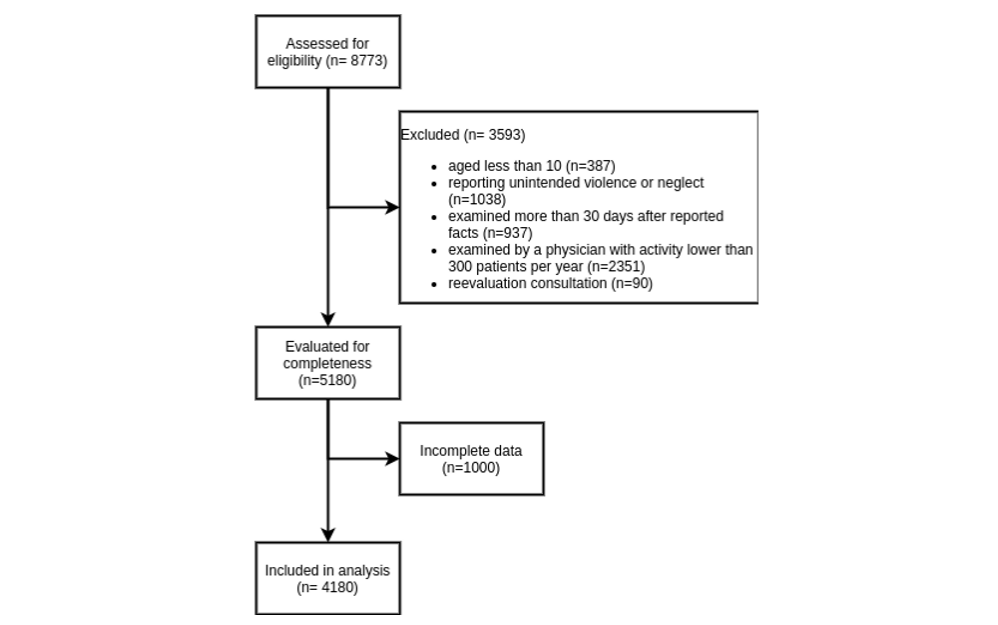
Flowchart of patients’ inclusion.

The flowchart of inclusions is shown in Figure 1. Complete and incomplete data were comparable in terms of age, sex, time to consultation, TIW, and all types of assaults (Table S1 in Multimedia Appendix 1).

### Determining a Typology of Situations of Violence

The optimal data set partition identified 5 profiles (see the “Identification of Homogeneous Profiles” section in Multimedia Appendix 1; also see Figures S1 and S2 in Multimedia Appendix 1). Table 1 presents the characteristics of each profile. Profile A included mostly individuals exposed to single assailants and low-impact assaults, resulting in lower levels of functional impairment (TIW median 3, IQR 1-4) and rarely causing physical injury (538/779, 69.1%, patients without any somatic traumatic lesions), with a majority being women (474/779, 60.9%). Profile B mainly included males exposed to violence (564/749, 75.3%) involving multiple (585/749, 78.1%) and often unknown (464/749, 61.9%) assailants. Profile C consisted primarily of situations involving police violence (567/719, 78.9%), involving young (median age 22, IQR 18-28 years) and predominantly male (687/719, 95.5%) patients held in police custody (678/719, 94.3%). Profile D comprised mainly individuals exposed to single assailants and high-impact assaults, resulting in higher levels of functional impairment (TIW median 4 days, IQR 3-6 days) and systematic physical injury (1092/1092, 100%, patients with somatic lesions), with a majority being men (852/1092, 78.0%). Profile E included mostly women (703/841, 83.6%) who were exposed to repeated assaults (679/841, 80.7%) perpetrated by intimate partners (678/841, 80.6%).

**Table 1:** Clinical categories underlying automatically determined profiles of situations of violence^a^.

Characteristics		A (n=779)	B (n=749)	C (n=719)	D (n=1092)	E (n=841)	All (n=4180)
**Patient characteristics**							
	Male gender, n (%)	305 (39.1)	564 (75.3)	687 (95.5)	852 (78.0)	138 (16.4)	2546 (60.9)
	Age (years), median (IQR)	32 (23-41)	29 (19-39)	22 (18-28)	33 (25-43)	31 (26-40)	30 (21-39)
	In police custody during consultation, n (%)	70 (9.0)	98 (13.1)	678 (94.3)	106 (9.7)	68 (8.1)	1020 (24)
	Time to consultation, median (IQR)	48 (24-81)	48 (24-72)	5 (3-9)	48 (24-72)	48 (24-72)	48 (13-72)
	TIW^b^, median (IQR)	3 (1-4)	4 (3-6)	2 (1-3)	4 (2-6)	4 (3-6)	3 (2-5)
**Assault characteristics, n (%)**							
	Workplace violence	122 (15.7)	45 (6.0)	4 (0.6)	104 (9.5)	9 (1.1)	284 (6.8)
	Intimate partner violence	76 (9.8)	9 (1.2)	30 (4.2)	15 (1.4)	678 (80.6)	808 (19.3)
	Intrafamily violence	73 (9.4)	44 (5.9)	34 (4.7)	37 (3.4)	105 (12.5)	293 (7.0)
	Police violence	11 (1.4)	25 (3.3)	567 (78.9)	5 (0.5)	0 (0)	608 (14.5)
	Violence against police officers	62 (8.0)	55 (7.3)	4 (0.6)	114 (10.4)	0 (0)	235 (5.6)
	Violent robbery	43 (5.5)	75 (10.0)	0 (0)	33 (3.0)	1 (0.1)	152 (3.6)
	Other types of violence	327 (42.0)	78 (10.4)	53 (7.4)	752 (68.9)	43 (5.1)	1253 (30.0)
**Physical trauma, n (%)**							
	Osteoarticular traumatic lesions	77 (9.9)	82 (10.9)	22 (3.1)	131 (12.0)	43 (5.1)	355 (8.5)
	Wounds	61 (7.8)	574 (76.6)	505 (70.2)	803 (73.5)	503 (59.8)	2446 (58.5)
	Bruises or hematomas	116 (14.9)	575 (76.8)	513 (71.3)	843 (77.2)	631 (75.0)	2678 (64.1)
	No somatic lesion	538 (69.1)	22 (2.9)	51 (7.1)	0 (0)	54 (6.4)	665 (15.9)
**Assailants characteristics, n (%)**							
	Classmate	19 (2.4)	10 (1.3)	0 (0)	36 (3.3)	3 (0.4)	68 (1.6)
	Co-worker	25 (3.2)	12 (1.6)	4 (0.6)	31 (2.8)	4 (0.5)	76 (1.8)
	Intimate partner	84 (10.8)	11 (1.5)	34 (4.7)	17 (1.6)	699 (83.1)	845 (20.2)
	Family member	81 (10.4)	41 (5.5)	33 (4.6)	46 (4.2)	99 (11.8)	300 (7.2)
	Unknown assailant(s)	356 (45.7)	464 (61.9)	30 (4.2)	570 (52.2)	0 (0)	1420 (34.0)
	Police officer	11 (1.4)	20 (2.7)	560 (77.9)	1 (0.1)	0 (0)	592 (14.2)
	Neighbor	38 (4.9)	33 (4.4)	14 (1.9)	84 (7.7)	14 (1.7)	183 (4.4)
	Nonspecific assailant	15 (1.9)	6 (0.8)	14 (1.9)	29 (2.7)	0 (0)	64 (1.5)
	Other known assailant	150 (19.3)	152 (20.3)	30 (4.2)	278 (25.5)	22 (2.6)	632 (15.1)
**Assault locations, n (%)**							
	Shopping center or shop	18 (2.3)	20 (2.7)	12 (1.7)	50 (4.6)	3 (0.4)	103 (2.5)
	Domicile of the assailant	12 (1.5)	10 (1.3)	3 (0.4)	18 (1.6)	16 (1.9)	59 (1.4)
	Conjugal home	32 (4.1)	1 (0.1)	18 (2.5)	3 (0.3)	378 (44.9)	432 (10.3)
	Family home	73 (9.4)	30 (4.0)	30 (4.2)	31 (2.8)	148 (17.6)	312 (7.5)
	Domicile of the affected individual	80 (10.3)	41 (5.5)	38 (5.3)	93 (8.5)	129 (15.3)	381 (9.1)
	School^c^	15 (1.9)	6 (0.8)	1 (0.1)	31 (2.8)	0 (0)	53 (1.3)
	Detention^d^	5 (0.6)	6 (0.8)	59 (8.2)	12 (1.1)	0 (0)	82 (2.0)
	Workplace	125 (16.1)	61 (8.1)	6 (0.8)	116 (10.6)	10 (1.2)	318 (7.6)
	Public building	55 (7.1)	54 (7.2)	60 (8.3)	104 (9.5)	18 (2.1)	291 (7.0)
	Common area of a residential complex	44 (5.7)	41 (5.5)	38 (5.3)	92 (8.4)	30 (3.6)	245 (5.9)
	Public transportation	27 (3.5)	20 (2.7)	12 (1.7)	33 (3.0)	2 (0.2)	94 (2.2)
	Private vehicle	5 (0.6)	1 (0.1)	8 (1.1)	8 (0.7)	7 (0.8)	29 (0.7)
	Street assault	272 (34.9)	451 (60.2)	408 (56.7)	466 (42.7)	78 (9.3)	1675 (40.1)
	Other type of location	16 (2.1)	7 (0.9)	26 (3.6)	35 (3.2)	22 (2.6)	106 (2.5)
**Assault characteristics, n (%)**							
	Multiple assailants	132 (16.9)	585 (78.1)	249 (34.6)	92 (8.4)	19 (2.3)	1077 (25.8)
	Weapon threats	38 (4.9)	42 (5.6)	25 (3.5)	49 (4.5)	40 (4.8)	194 (4.6)
	Weapon usage	75 (9.6)	159 (21.2)	131 (18.2)	199 (18.2)	107 (12.7)	671 (16.1)
	Beating the face	201 (25.8)	498 (66.5)	295 (41.0)	715 (65.5)	559 (66.5)	2268 (54.3)
	Blows provoking the fall	137 (17.6)	541 (72.2)	236 (32.8)	251 (23.0)	223 (26.5)	1388 (33.2)
	Knocked on the ground	13 (1.7)	135 (18.0)	67 (9.3)	50 (4.6)	55 (6.5)	320 (7.7)
	Repeated assault	144 (18.5)	68 (9.1)	35 (4.9)	92 (8.4)	679 (80.7)	1018 (24.4)
	Sequestration	2 (0.3)	7 (0.9)	0 (0)	7 (0.6)	4 (0.5)	20 (0.5)

^a^Controlled observational study of 4180 individuals who experienced assaults who attended a medical examination during the year 2015 in a French department of forensic medicine (Bondy, Greater Paris area). Data were drawn from electronic health records. A, B, C, D, and E are profiles of violent situations determined by identifying homogeneous groups of patients using Partitioning Around Medoids, a clustering algorithm. Similarity between patients was based on multiple variables that defined a violent situation; these variables are described above in each profile and in the overall population.

^b^TIW: total incapacity to work.

^c^Any level of formation.

^d^Police station or during police transportation.

Scales for each profile and patient-reported symptoms related to psychological trauma are described in Table 2. Self-rated scales had higher scores at the time of the assault compared with the consultation time (Table 2). Women who experienced repeated IPV (profile E) showed high scores for fear during both the assault (median 6, IQR 4-6) and consultation (median 5, IQR 2-6), as well as for the perception of a life threat during the assault (median 4, IQR 0-6) and consultation (median 2, IQR 0-6). Individuals exposed to violence involving multiple assailants (profile B) had high scores for the perception of a life threat during the assault (median 3, IQR 0-6). Young males exposed to police violence (profile C) reported low scores for pain during the assault (median 3, IQR 2-5) and low scores for fear both during the assault (median 3, IQR 0-5) and during the consultation (median 0, IQR 0-3). Women experiencing low-impact assaults (profile A) reported low scores for pain during the consultation (median 2, IQR 0-4).

**Table 2:** Highlighting patterns of psychological trauma associated with different profiles of situations of violence^a,b^.

Characteristics		A (n=779)	B (n=749)	C (n=719)	D (n=1092)	E (n=841)	All (n=4180)
**Scale characteristics, median (IQR)**							
	Pain (assault)	4 (2-5)	4 (3-5)	3 (2-5)	4 (3-5)	4 (3-5)	4 (3-5)
	Pain (consultation)	2 (0-4)	3 (2-4)	3 (1-4)	3 (2-4)	3 (2-4)	3 (2-4)
	Fear (assault)	5 (2-6)	5 (2-6)	3 (0-5)	5 (0-6)	6 (4-6)	5 (1-6)
	Fear (consultation)	3 (0-6)	3 (0-5)	0 (0-3)	3 (0-5)	5 (2-6)	3 (0-5)
	Perception of a life threat (assault)	0 (0-6)	3 (0-6)	0 (0-3)	0 (0-6)	4 (0-6)	0 (0-6)
	Perception of a life threat (consultation)	0 (0-4)	0 (0-4)	0 (0-0)	0 (0-4)	2 (0-6)	0 (0-4)
	Functional impairment	1 (0-2)	2 (0-2)	0 (0-1)	1 (0-2)	1 (0-2)	1 (0-2)
	Quality of interaction between patient and physician	5 (5-6)	5 (5-6)	5 (4-6)	5 (5-6]	5 (5-6)	5 (5-6)
	Psychosomatic index	3 (1-5)	3 (1-3)	1 (0-3)	3 (1-3)	3 (3-4)	3 (1-3)
**Psychological trauma symptoms, n (%)**							
	Sleeping disorder symptoms (difficulty initiating sleep, frequent awakenings, early-morning awakening)	58 (7.4)	69 (9.2)	5 (0.7)	78 (7.1)	95 (11.3)	305 (7.3)
	Loss of appetite	35 (4.5)	36 (4.8)	2 (0.3)	34 (3.1)	60 (7.1)	167 (4.0)
	Stress symptoms (recurrent memories, avoidance, hypervigilance)	76 (9.8)	77 (10.3)	9 (1.3)	93 (8.5)	79 (9.4)	334 (8.0)
	Pain	219 (28.1)	281 (37.5)	205 (28.5)	339 (31.0)	278 (33.1)	1322 (31.6)
	Fear	89 (11.4)	93 (12.4)	10 (1.4)	87 (8.0)	115 (13.7)	394 (9.4)

^a^Controlled observational study of 4180 individuals who experienced assaults who attended a medical examination during the year 2015 in a French department of forensic medicine (Bondy, Greater Paris area). Data were drawn from electronic health records.

^b^For each profile of situations of violence, we reported the median scores and IQR of scales quantifying subjective elements and in parallel related patients’ symptoms.

We found significant differences (*P<*.001) in median ratings between physicians for each scale (Table S6 in Multimedia Appendix 1). Results for pain during the assault, comparing pairs of physicians in the overall population and within profiles of situations involving violence, are shown in Multimedia Appendix 2 (also see Table S3 in Multimedia Appendix 1). All physicians had examined more than 30 patients in each profile of situations involving violence (Table S2 in Multimedia Appendix 1). For pain during the assault, we observed significant differences between 16 out of 45 pairs of physicians in the overall population, while fewer than 4 pairs of physicians showed significant differences when assessed within profiles of situations involving violence (Multimedia Appendix 2). Results were similar for other self-rated scales, but most pairwise comparisons remained significant for physician-rated scales (Table S3 in Multimedia Appendix 1).

All pain, fear, and life threat scales were significantly associated with functional impairment as measured by TIW (Table 3).

**Table 3:** Association of scale ratings with functional impairment^a^.

Scales	β (95% CI)
Pain (assault)	0.68 (0.57 to 0.80)
Pain (consultation)	0.72 (0.59 to 0.85)
Fear (assault)	0.33 (0.24 to 0.42)
Fear (consultation)	0.42 (0.33 to 0.51)
Perception of a life threat (assault)	0.33 (0.25 to 0.41)
Perception of a life threat (consultation)	0.37 (0.27 to 0.46)
Functional impairment	2.65 (2.49 to 2.81)
Quality of interaction between patient and physician	0.46 (0.20 to 0.72)
Psychosomatic index	0.09 (–0.04 to 0.23)

^a^Controlled observational study of 4180 individuals who experienced assaults who attended a medical examination during the year 2015 in a French department of forensic medicine (Bondy, Greater Paris area). Data were drawn from electronic health records. We assessed the association of each scale with functional impairment using univariate linear regression models. Linear regression coefficients (betas) and their 95% CIs are reported. For example, the fear during assault coefficient is 0.33; thus, on average, 3 points of fear during assault is associated with 1 extra day of functional impairment as days of total incapacity to work.

### Reproducibility of Scales in the Same Violent Situation Profile

#### Interrater Reproducibility

Interrater reproducibility of scales was mild to good within the same violent situation profiles. Results for all scales are presented in Tables S4 and S5 in Multimedia Appendix 1. Kendall *W* ranged from 0.43 (pain during assaults, paired physicians 7 and 9) to 0.66 (psychosomatic index, paired physicians 6 and 8).

#### Intrarater Reproducibility

Intrarater reproducibility of scales within the same violent situation profiles was found to be mild to good (Table S7 in Multimedia Appendix 1). The Kendall coefficient of concordance ranged from 0.46 (perception of a life threat during the assault, physicians 3 and 7) to 0.66 (psychosomatic index, physician 8).

## Discussion

### Principal Findings

In this study, we investigated tools for quantifying subjective elements related to functional impairment in individuals exposed to violence, based on a typology of situations involving violence derived from massively multivariate data. A clustering algorithm identified 5 remarkably stable profiles of violent situations, each with comparable sizes and characteristics consistent with clinical practice. This typology allowed us to evaluate the subjective scales with the assumption that within each type, the characteristics of violence and exposed individuals were homogeneous. Consequently, any remaining variability in scale results could reasonably be attributed to inter- and intrarater performance. All pain, fear, and life threat scales were significantly associated with functional impairment, indicating that they effectively measure elements contributing to the overall assessment of functional impairment. These scales highlighted different patterns of psychological trauma associated with the various profiles of situations involving violence. There were a few significant differences in ratings between physicians for self-rated scales within the same violent situation profiles. Finally, we found mild to good inter- and intrarater reproducibility for all scales within the same violent situation profiles.

Patients were clustered consistently within intuitive clinical categories (Table 1). Differences in scale scores between violent situation profiles should be examined in light of these clinical aspects (Table 2). First, individuals exposed to violence involving multiple assailants (profile B) more frequently reported a perception of a life threat during the assault, which aligns with the high intensity of these assaults, often involving blows that caused falls (541/749, 72.2%) or being knocked to the ground (135/749, 18%). Interestingly, while the perception of a life threat was higher during the assault, it did not seem to persist over time and was lower at the time of consultation. Women exposed to IPV (profile E) displayed a different pattern of fear and perception of a life threat, with higher scores recorded both during the assault and at the consultation. This persistence of symptoms is consistent with these patients experiencing mostly repeated assaults (679/841, 80.7%) by known, intimate assailants, which increases the credibility and fear of future attacks. The scales were consistent with patients’ reported symptoms such as fear, sleeping disorders, and loss of appetite, while providing richer and more comprehensible information. Second, young men held in police custody and reporting police violence (profile C) exhibited lower scores for fear both during the assault and at the consultation, as well as a lower score for pain during the assault (Table 2). These lower scores may reflect underlying psychosocial behaviors of pain or fear denial in this population. These findings are consistent with their lower functional impairment measurements (median 2, IQR 1-3 days of TIW). However, it has been previously shown [17] that their functional impairment was systematically evaluated at lower values and that they were examined in shorter intervals after the assault (median 5, IQR 3-9 hours) and under specific circumstances (in police custody).

There were differences in ratings between physicians in the overall population (Table S6 in Multimedia Appendix 1) for all scales and for most pairs of physicians (Table S3 in Multimedia Appendix 1). However, because all physicians were instructed to ask the questions in the same manner (see the “Questions Asked During Consultations” section in Multimedia Appendix 1), these observed differences were likely attributable to variations in the situations involving violence. For instance, some physicians more frequently consulted patients from cluster A than cluster D, possibly due to planning reasons or personal preferences. Supporting this interpretation, we found that within the same profiles of situations of violence, only a few significant differences for self-rated scales remained (Table S3 in Multimedia Appendix 1 and Multimedia Appendix 2).

Self-rated scales were associated with functional impairment as measured by TIW; specifically, TIW increased with higher scale scores. This association underscores the clinical relevance of these scales, as subjective elements reported by patients themselves were directly linked to TIW, which is otherwise solely determined by physician assessment. These scales, now shown to be associated with functional impairment, could provide standardized access to a patient’s perspective and enhance clinical examination.

The reproducibility of the scales within the same profiles of situations of violence was found to be mild to good, ranging from 0.43 to 0.66 for interrater and 0.46 to 0.66 for intrarater reproducibility across the 5 situations of violence profiles. Higher reproducibility (>70%) can be achieved with similar scales [11] in a classic design, such as when patients are evaluated at a 2-week interval using the same scales. These differences could arise from evaluating patients in real-life conditions rather than in the controlled environment of a research study. Additionally, our study design made it challenging to determine whether strong specificities of individual situations persisted among matched patients. Such remaining specificities could lead to systematic differences in their scale scores, thereby reducing reproducibility. Increasing the number of situations of violence profiles helped test this hypothesis. As the number of profiles grew, the remaining specificities among patients within the same profiles decreased. A sensitivity analysis revealed slightly higher reproducibility values when differentiating 800 profiles (Tables S5 and S7 in Multimedia Appendix 1). This suggests that the reproducibility results were not underestimated and that the situations of violence profiles were sufficiently detailed.

Reproducibility was assessed across similar situations involving violence rather than individual patients seen at different time intervals to stay close to real-life conditions, as a classic design was not feasible in this context. First, a classic design could not assess the reproducibility of subjective elements at consultations because a first consultation for violence cannot be replicated. Second, if 2 consultations occurred close together, patients might remember their responses for subjective elements during the assault, whereas if consultations were distant, memories might fade, affecting the accuracy of their responses.

This study has several shortcomings. First, as a monocentric study, it may not fully represent forensic practice across the country. However, the population served by the study is among the most diverse in France in terms of geographical origins, socioeconomic status, and culture, according to the National Institute for Statistics and Economic Studies (Insee). Second, many patients (1000/5180, 19.31%) were excluded due to missing data. However, missing data were comparable for baseline characteristics. Third, combining physical and psychological impairments poses a challenge, and we may have lacked the power to detect small effect sizes. Finally, our definition of situations involving violence could have been enhanced by including information related to psychological trauma. However, psychological trauma symptoms were not systematically reported by physicians and could neither be reliably used nor provide informative data for determining the typology.

To our knowledge, this study is the first to investigate the quantification of subjective elements related to functional impairment in individuals exposed to violence within the context of daily medical examinations in a judicial setting. Although the study is based on data collected 8 years ago, it remains relevant and current, as the activity of the forensic medicine department has not significantly changed since 2015, neither in volume nor in the reasons for the examination. The missions remain the same, and the practices have not significantly evolved in either direction. No modifications to the French penal code that could affect the relevance of this study have been noted either. Quantification of subjective elements provided direct access to patients’ opinions in a synthetic and reliable manner and revealed significant differences between situations involving violence, such as variations in fear and perception of a life threat among those exposed to IPV. Further studies are planned to explore whether these tools can lead to straightforward interpretations by magistrates and judicial police officers and to assess their impact on judicial decisions.

### Conclusions

The pain, fear, and life threat scales were correlated with higher functional impairment, aligning with expert knowledge, and demonstrated fair reproducibility in real-life conditions for similar situations of violence. Subjective elements related to functional impairment in individuals exposed to violence can be quantified using Likert scales during medical interviews. High scores in fear and perception of a life threat, both during the assault and at the medical consultation, suggest greater impairment related to psychological trauma, particularly following IPV.

## Appendix

Multimedia Appendix 1 Additional data analysis.

Multimedia Appendix 2 Differences in ratings of subjective elements between physicians in the same profile of situations of violence.

## Abbreviations

ICF: International Classification of Functioning, Disability, and Health
IPV: intimate partner violence
TIW: total incapacity to work
WHO: World Health Organization

## Data Availability

The data sets generated or analyzed during the study are not publicly available due to the fact they are considered sensitive personal data in the sense of the General Data Protection Regulation (GDPR) but are available from the corresponding author on reasonable request and if compliant with GDPR rules.

## References

[1] Krug EG, Mercy JA, Dahlberg LL, Zwi AB (2002). The world report on violence and health. Lancet.

[2] Stöckl H, Devries K, Rotstein A, Abrahams N, Campbell J, Watts C, Moreno CG (2013). The global prevalence of intimate partner homicide: a systematic review. Lancet.

[3] Black MC, Basile KC, Breiding MJ, Smith SG, Walters ML, Merrick MT, Chen J, Stevens MR (2011). National intimate partner and sexual violence survey: 2010 summary report. US Department of Justice: Office of Justice Programs.

[4] Scarpa A (2003). Community violence exposure in young adults. Trauma Violence Abuse.

[5] Gignon M, Paupière S, Jardè O, Manaouil C (2010). Victims of assault: a Europe-wide review of procedures for evaluating the seriousness of injuries. Med Sci Law.

[6] Guez S, Laugier V, Saas C, Lefèvre T, Julia G (2020). L'IA, le légiste et le magistrat. Le projet big data drop It et l'exemple du traitement médicolégal des violences interpersonnelles. Sciences et Sens de l'intelligence Artificielle.

[7] Johansen VA, Wahl AK, Eilertsen DE, Weisaeth L, Hanestad BR (2007). The predictive value of post-traumatic stress disorder symptoms for quality of life: a longitudinal study of physically injured victims of non-domestic violence. Health Qual Life Outcomes.

[8] Resnick HS, Acierno R, Kilpatrick DG (1997). Health impact of interpersonal violence. 2: medical and mental health outcomes. Behav Med.

[9] Kilpatrick DG, Ruggiero KJ, Acierno R, Saunders BE, Resnick HS, Best CL (2003). Violence and risk of PTSD, major depression, substance abuse/dependence, and comorbidity: results from the National Survey of Adolescents. J Consult Clin Psychol.

[10] Kilpatrick DG, Acierno R (2005). Mental health needs of crime victims: epidemiology and outcomes. Journal of Traumatic Stress.

[11] Hawker GA, Mian S, Kendzerska T, French M (2011). Measures of adult pain: Visual Analog Scale for Pain (VAS Pain), Numeric Rating Scale for Pain (NRS Pain), McGill Pain Questionnaire (MPQ), Short-Form McGill Pain Questionnaire (SF-MPQ), Chronic Pain Grade Scale (CPGS), Short Form-36 Bodily Pain Scale (SF-36 BPS), and Measure of Intermittent and Constant Osteoarthritis Pain (ICOAP). Arthritis Care Res (Hoboken).

[12] Graf C (2008). The Lawton instrumental activities of daily living scale. Am J Nurs.

[13] Chariot P, Lefèvre Thomas, Denis Céline, Lepresle Aude (2015). [The somatopsychic index, a tool devoted to a practical appraisal of functional impairment among victims of assaults]. Presse Med.

[14] Wilkerson M, Hayes D Neil (2010). ConsensusClusterPlus: a class discovery tool with confidence assessments and item tracking. Bioinformatics.

[15] Pohlert T (2014). Calculate Pairwise Multiple Comparisons of Mean Rank Sums (PMCMR). The Comprehensive R Archive Network.

[16] Matthias G, Lemon J, Fellows I, Singh P (2012). Various coefficients of interrater reliability and agreement (R package version 0.84). The Comprehensive R Archive Network.

[17] Lefèvre Thomas, Briffa H, Thomas G, Chariot P (2012). Evaluating the functional impairment of assault survivors in a judicial context - a retrospective study. J Forensic Leg Med.

[18] Chariot P, Bécache Nathalie, François-Purssell Irène, Dantchev Nicolas, Delpla Pierre-André, Fournier Lionel, Proust Bernard (2013). [Evaluating the total incapacity to work: implementing French National Authority for Health guidelines in clinical practice]. Presse Med.

[19] Lefèvre T, Chauvin P (2015). A general framework for a reliable multivariate analysis and pattern recognition in high-dimensional epidemiological data, based on cluster robustness: a tutorial to enrich the epidemiologists' toolkit. Rev Epidemiol Sante Publique.

[20] Kaufman L, Rousseeuw PJ (2009). Finding Groups in Data: An Introduction to Cluster Analysis.

[21] Monti S, Tamayo P, Mesirov J, Golub T (2003). Consensus clustering: a resampling-based method for class discovery and visualization of gene expression microarray data. Mach Learn.

[22] Hollander M, Wolfe DA, Chicken E (2013). Nonparametric Statistical Methods.

[23] Conover W, Iman R.L. (1979). On multiple-comparisons procedures (Los Alamos Scientific Laboratory technical report number LA-7677-MS). OSTI.

[24] Kendall MG, Smith BB (1939). The problem of m rankings. Ann Math Stat.

[25] Legendre P (2005). Species associations: the Kendall coefficient of concordance revisited. JABES.

