# Supplementary material for: Functional Impairment in Individuals Exposed to Violence Based on Electronical Forensic Medical Record Mining and Their Profile Identification: Controlled Observational Study
Source: JMIR Public Health Surveill. 2024 Sep 27;10:e43563. doi: 10.2196/43563 (PMC11470214; doi:10.2196/43563)
Supplement: Multimedia Appendix 1 [file publichealth_v10i1e43563_app1.docx]

**Supplementary materials**

*1. Inclusion and exclusion criteria*

Patients reporting deliberate assault and battery were assessed for eligibility. We excluded patients aged less than 10, patients reporting unintended violence or neglect, patients examined more than 30 days after reported facts, patients examined by a physician with activity lower than 300 patients per year, and patients examined for a second assessment of their functional impairment. We only considered patients without any missing data in the analysis.

*2. Data collection*

All data was extracted from an Access database, which was systematically built from EHR medical reports by collecting variables via a standardized form.

*3 Description of the scales*

We introduced three self-evaluated Likert scales to measure the expression of pain, fear and the perception of a life threat at two distinct endpoints: at the time of the assault and during consultation. Each Likert item had 7 levels (from 0 to 6). The physician asked the question in a general manner and was free to repeat it twice, while he presented a graduated scale for visual aid, that the patient rated himself. For the pain scale the physician asked (translated from French): “On a scale of 0 to 6 and knowing that 0 means no pain and 6 is the maximum amount of pain you can imagine, can you rate how much pain you experienced during the assault?”, and then “Similarly, on a scale of 0 to 6 and knowing that 0 means no pain and 6 is the maximum amount of pain you can imagine, can you rate how much pain you are experiencing now?”. Other questions for fear and perception of a life threat were asked similarly (see supplementary materials section 7).

We introduced two physician-evaluated Likert scales, with 7 levels from 0 to 6. One aimed at measuring the intensity of the global functional impairment at the time of the consultation. Physicians were asked to rate the intensity from 0 to 6 (0 meaning no functional impairment and 6 a maximum functional impairment). The other scale aimed at measuring quality of interaction between the physician and the patient. Physicians were asked to rate from 0 to 6 (0 being the worse quality of interaction and 6 the most satisfying).

Finally we introduced the psychosomatic index, a 7 levels index from 0 to 6 which physicians were asked to rate from 0 if physical trauma was the only source of functional impairment to 6 if psychological trauma was the only source of functional impairment [(12)](https://paperpile.com/c/L7lM1n/ToeC).

*4 Defining violence situations*

Individuals exposed to violence were characterized by age, gender and if they were held in police custody at the time of consultation. Assailants were characterized within nine descriptive categories. Assaults were characterized by the type of assaults (7 categories), type of assaults places (14 categories), time elapsed between assault and consultation, other assault circumstances and assault outcomes. Other assault circumstances corresponded to the presence or absence of aggravating factors: weapon threat, weapon usage, face beating, blows provoking the fall, to be knocked while on the ground, repeated assault, sequestration, and multiple assailants. Assaults outcomes were characterized by physical trauma and functional impairment. Functional impairment was quantified as requested by French judicial authorities in days of total incapacity to work (TIW) [(13,14)](https://paperpile.com/c/L7lM1n/7Src+rzQJ). TIW measures the total functional impairment induced by the assault whereas our scale of functional impairment only measures it at time of the consultation. Physical trauma measurements corresponded to the presence or absence of: any somatic lesion, bruises or hematomas, wounds, and osteo-articular traumatic lesions.

*5 Sensitivity analysis*

We conducted the same intra-rater and inter-rater reproducibility analysis while increasing the number of SoV profiles that the clustering algorithm could identify (200 and 800 profiles). Hence for example with 800 SoV profiles, patients matched within the same profile were more homogeneous than with 5 profiles. It allowed us to test if our typology of SoV was not sufficiently fine grained and caused lower reproducibility results.

With 800 SoV profiles, inter-rater reproducibility results ranged between 0.42 (pain during consultation, paired physicians n°7 and 9) and 0.72 (functional impairment, paired physicians n° 6 and 9). Intra-rater reproducibility results ranged between 0.39 (perception of a life threat, physician n°1) and 0.76 (psychosomatic index, physician n°10). If we focus on pain during assault, inter-rater reproducibility results were slightly higher for most pairs of physicians (38 on 45) with 800 SoV profiles (ranging from 0.46 to 0.61) than with 5 SoV profiles (ranging from 0.43 to 0.56). In addition, 6 out of 7 of the pairs of physicians that did not increase were including the same physician (number 10). Pain during assault intra-rater reproducibility results were also slightly higher for most physicians (8 out of 10) with 800 SoV profiles (ranging from 0.48 to 0.64) than with 5 SoV profiles (ranging from 0.50 to 0.61).

*6 Software used for analyses*

Analyses were conducted on R version 3.3.2, under linux-Ubuntu 16.04 OS. We used the *Consensus Cluster Plus* package for clustering. We used the *PMCMR* package for Kruskall-Wallis post-hoc analysis. And we used the *irr* package for inter and intra-rater reproducibility.

*7 Questions asked during consultations*

Three self-evaluated scales (the physician asks the individual exposed to violence):

- Pain assessment: “On a scale of 0 to 6 and knowing that 0 means no pain and 6 is the maximum amount of pain you can imagine, can you rate how much pain you experienced during the assault?”, and then “Similarly, on a scale of 0 to 6 and knowing that 0 means no pain and 6 is the maximum amount of pain you can imagine, can you rate how much pain you are experiencing now?”.
- Fear assessment: “On a scale of 0 to 6 and knowing that 0 means no fear and 6 is the maximum amount of fear you can imagine, can you rate how much fear you experienced during the assault?”, and then “Similarly, on a scale of 0 to 6 and knowing that 0 means no pain and 6 is the maximum amount of fear you can imagine, can you rate how much fear you are experiencing now?”.
- Perception of a life threat: “On a scale of 0 to 6 and knowing that 0 means no perception of a life threat and 6 is the maximum amount of perception of a life threat you can imagine, can you rate how much perception of a life threat you experienced during the assault?”, and then “Similarly, on a scale of 0 to 6 and knowing that 0 means no perception of a life threat and 6 is the maximum amount of perception of a life threat you can imagine, can you rate how much perception of a life threat you are experiencing now?”.

Two physician-evaluated scales (the physician must answer the question):

- Global functional impairment at the time of the consultation: “On a scale of 0 to 6 and knowing that 0 means no functional impairment and 6 is full functional impairment, can you rate how much functional impairment the individual exposed to violence is experiencing now?”
- Quality of interaction: “On a scale of 0 to 6 and knowing that 0 means a low quality of interaction and 6 a good quality of interaction, can you rate the quality of interaction with the individual exposed to violence during the consultation?”

The psychosomatic index (the physician must answer the question): “Can you rate the relative part of psychological and physical trauma that you think contributed to functional impairment of this individual exposed to violence, knowing that 0 means that only physical trauma contributed to functional impairment whereas 6 means only psychological trauma contributed to it” (1)

*8 Kendall’s W coefficient of concordance*

S is a sum-of-squares statistic over the row sums of ranks R_i_.


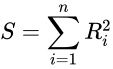


Kendall’s W statistic can be obtained from the following formula:


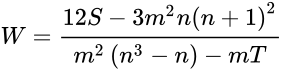


Where n is the number of objects and m is the number of variables. T is a correction factor for tied ranks:


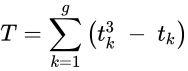


In which t_k_ is the number of tied ranks in each (k) of g groups of ties. The sum is computed over all groups of ties found in all m variables of the data table. T equal 0 when there are no tied values (2).

*9 Comparison of complete and incomplete data*

**Table S1. Comparisons between complete and incomplete data**

|  | Complete data  (n=4180) | Incomplete data  (n=1000) |
| --- | --- | --- |
| **Patient characteristics** | | |
| Age (years, median and IQR) | 30 [21-39] | 33 [24-43] |
| Male gender | 2546 (61%) | 624 (62%) |
| Time to consultation (hours, median and IQR) | 48 [13-72] | 48 [12-72] |
| TIW (days, median and IQR) | 3 [2-5] | 3 [1-5] |
| **Assault characteristics** | | |
| Workplace violence  Intra-family violence  Intimate partner violence  Police violence  Violence against police officers  Violent robbery  Other type of violence | 284 (7%)  293 (7%)  808 (19%)  608 (15%)  235 (6%)  152 (4%)  1253 (30%) | 46 (5%)  97 (10%)  208 (21%)  160 (16%)  16 (2%)  37 (4%)  281 (28%) |

Controlled observational study of 4180 assaults’ victims who attended a medical examination during the year 2015 in a French departement of forensic medicine (Bondy, Greater Paris area). Data drawn from electronical health records.

IQR: interquartile range, TIW: total incapacity to work

*10 Identification of homogeneous profiles*

In the *partition around medoid* algorithm, each cluster is defined as a group of points centered around a *medoid,* which itself is a point belonging to the dataset. *Medoids* are selected to optimize the reduction of a distance measure between them and the other clusters points.

We used the Gower distance to compute the distance between two points of our dataset. The Gower distance is defined as the average over: an interval-scaled absolute difference for each continuous variable, the proportion of disagreement for all nominal variables, the proportion of disagreement for all symmetric binary variables and the Jaccard distance for all asymmetric binary variables. Only gender was considered as a symmetric variable.

The consensus clustering framework allows computing for each pair of point consensus values which are ratios between the number of time a pair is assigned to the same cluster against the number of time they were both sampled together. Their distribution reflects the stability of the clusters assignments (Figure S1-2). We ran the consensus clustering algorithm with 100 iterations and a 80% subsampling ratio for 2 to 10 clusters

**Figure S1. Cumulative distribution function of consensus values as a function of the number of clusters**


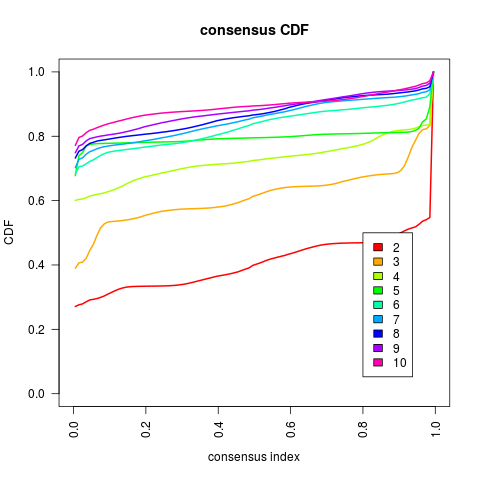


Controlled observational study of 4180 assaults’ victims who attended a medical examination during the year 2015 in a French departement of forensic medicine (Bondy, Greater Paris area). Data drawn from electronical health records.

Cumulative distribution functions (CDFs) of consensus values are plotted for each number of clusters. The number of clusters is represented by a color code. The flatter is the CDF between 0.1 and 0.9, the more bimodal is the distribution of consensus values and the more stable are the cluster assignments. The flattest CDF is obtained for 5 clusters.

**Figure S2. Matrices of consensus values for selecting the optimal number of clusters according to a group robustness approach**

**
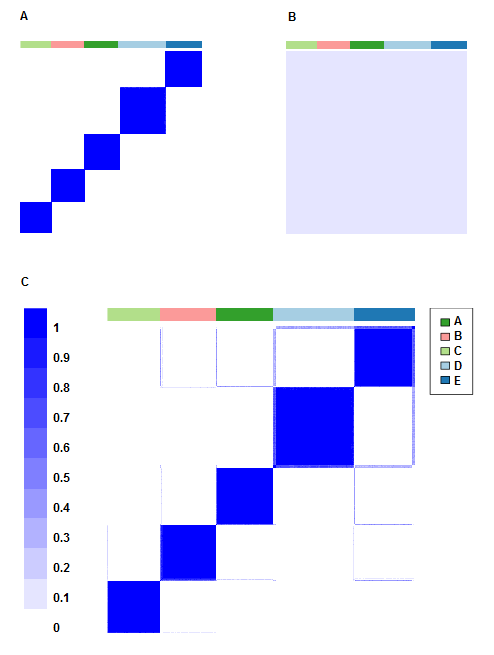
**

Controlled observational study of 4180 assaults’ victims who attended a medical examination during the year 2015 in a French departement of forensic medicine (Bondy, Greater Paris area). Data drawn from electronical health records.

A consensus matrix represents consensus values for all possible pairs of points in the dataset. Points of the dataset are rearranged similarly in lines and columns so that pairs that have high consensus values become close neighbors. Hence diagonal submatrices show high values of consensus and correspond to stable clusters. Panel **A** shows the example of a consensus matrix for a dataset including 5 perfectly separated clusters. Panel **B** shows the example of a consensus matrix for a noisy dataset not including any clusters. Panel **C** shows the consensus matrix of our dataset for 5 clusters. The color gradient codes for 100% consensus for deep blue versus 0% consensus for white.

11 Kruskall-Wallis pairwise post-hoc analyses

**Table S2. Number of physicians per clusters - N(%)**

|  | **A** | **B** | **C** | **D** | **E** |
| --- | --- | --- | --- | --- | --- |
|  | n= 779 | n= 749 | n= 719 | n= 1092 | n= 841 |
| **1** | 47 (6) | 42 (6) | 31 (4) | 59 (5) | 55 (7) |
| **2** | 89 (11) | 78 (10) | 53 (7) | 101 (9) | 109 (13) |
| **3** | 84 (11) | 65 (9) | 65 (9) | 125 (11) | 88 (10) |
| **4** | 87 (11) | 143 (19) | 163 (23) | 216 (20) | 136 (16) |
| **5** | 68 (9) | 61 (8) | 81 (11) | 108 (10) | 55 (7) |
| **6** | 57 (7) | 62 (8) | 49 (7) | 74 (7) | 74 (9) |
| **7** | 106 (14) | 46 (6) | 82 (11) | 79 (7) | 51 (6) |
| **8** | 103 (13) | 125 (17) | 77 (11) | 138 (13) | 129 (15) |
| **9** | 60 (8) | 55 (7) | 55 (8) | 73 (7) | 60 (7) |
| **10** | 78 (10) | 72 (10) | 63 (9) | 119 (11) | 84 (10) |

Controlled observational study of 4180 assaults’ victims who attended a medical examination during the year 2015 in a French departement of forensic medicine (Bondy, Greater Paris area). Data drawn from electronical health records.

**Table S3. Post-hoc analysis of all scales in all populations and within clusters.**

|||||: p-value < 0.05, -----: p-value > 0.05 for Conover post-hoc analysis of differences in median between pair a pair physicians

Pain during assault

1 2 3 4 5 6 7 8 9

2 ||||

3 ----- ||||

4 ----- |||| -----

5 ----- |||| |||| -----

6 |||| ----- ----- ----- ||||

7 ----- |||| ----- ----- ----- ||||

8 |||| ----- ----- ----- |||| ----- ||||

9 ----- |||| ----- ----- ----- ----- ----- -----

10 ----- |||| ----- ----- ----- |||| ----- |||| -----

#################################################################

Profile A

1 2 3 4 5 6 7 8 9

2 -----

3 ----- -----

4 ----- ----- -----

5 ----- |||| ----- -----

6 ----- ----- ----- ----- -----

7 ----- ----- ----- ----- ----- -----

8 ----- ----- ----- ----- ----- ----- -----

9 ----- ----- ----- ----- ----- ----- ----- -----

10 ----- ----- ----- ----- ----- ----- ----- ----- -----

Profile B

1 2 3 4 5 6 7 8 9

2 ||||

3 ----- -----

4 ----- ----- -----

5 ----- ----- ----- -----

6 ----- ----- ----- ----- -----

7 ----- ----- ----- ----- ----- -----

8 ----- ----- ----- ----- ----- ----- -----

9 ----- ----- ----- ----- ----- ----- ----- -----

10 ----- ----- ----- ----- ----- ----- ----- ----- -----

Profile C

1 2 3 4 5 6 7 8 9

2 -----

3 ----- -----

4 ----- ----- -----

5 ----- ----- ----- -----

6 ----- ----- ----- ----- -----

7 ----- ----- ----- ----- ----- -----

8 |||| ----- ----- |||| |||| ----- ||||

9 ----- ----- ----- ----- ----- ----- ----- -----

10 ----- ----- ----- ----- ----- ----- ----- ----- -----

Profile D

1 2 3 4 5 6 7 8 9

2 ||||

3 ----- ||||

4 ----- |||| -----

5 ----- ----- ----- -----

6 ----- ----- ----- ----- -----

7 ----- ----- ----- ----- ----- -----

8 ----- ----- ----- ----- ----- ----- -----

9 ----- ----- ----- ----- ----- ----- ----- -----

10 ----- |||| ----- ----- ----- ----- ----- ----- -----

Profile E

1 2 3 4 5 6 7 8 9

2 -----

3 ----- -----

4 ----- ----- -----

5 ----- |||| ----- -----

6 ----- ----- ----- ----- -----

7 ----- ----- ----- ----- ----- -----

8 ----- ----- ----- ----- |||| ----- -----

9 ----- ----- ----- ----- ----- ----- ----- -----

10 ----- ----- ----- ----- ----- ----- ----- ----- -----

#################################################################

Pain during consultation

1 2 3 4 5 6 7 8 9

2 ||||

3 |||| ||||

4 |||| |||| ||||

5 |||| ----- ----- ||||

6 |||| ----- |||| |||| -----

7 ----- |||| ----- |||| ----- ||||

8 |||| ----- ----- |||| ----- ----- -----

9 ----- |||| ----- |||| ----- |||| ----- ||||

10 |||| ----- |||| |||| |||| ----- |||| ----- ||||

#################################################################

Profile A

1 2 3 4 5 6 7 8 9

2 -----

3 ----- -----

4 |||| ----- -----

5 ----- ----- ----- ||||

6 |||| ----- ----- ----- -----

7 ----- ----- ----- ----- ----- -----

8 ----- ----- ----- ----- ----- ----- -----

9 ----- ----- ----- |||| ----- |||| ----- -----

10 ----- ----- ----- ----- ----- ----- ----- ----- -----

Profile B

1 2 3 4 5 6 7 8 9

2 ||||

3 ----- -----

4 |||| ----- ||||

5 ----- ----- ----- ||||

6 |||| ----- ----- ----- -----

7 ----- ----- ----- |||| ----- -----

8 ----- ----- ----- |||| ----- ----- -----

9 ----- ----- ----- |||| ----- ----- ----- -----

10 ----- ----- ----- ----- ----- ----- ----- ----- -----

Profile C

1 2 3 4 5 6 7 8 9

2 -----

3 ----- -----

4 |||| ----- -----

5 ----- ----- ----- -----

6 ----- ----- ----- ----- -----

7 ----- ----- ----- |||| ----- -----

8 ----- ----- ----- ----- ----- ----- -----

9 ----- ----- ----- |||| ----- ----- ----- -----

10 |||| ----- ----- ----- ----- ----- |||| ----- ||||

Profile D

1 2 3 4 5 6 7 8 9

2 -----

3 ----- -----

4 |||| ----- ||||

5 ----- ----- ----- -----

6 ----- ----- ----- ----- -----

7 ----- ----- ----- ----- ----- -----

8 ----- ----- ----- ----- ----- ----- -----

9 ----- |||| ----- |||| ----- ----- ----- ||||

10 ----- ----- ----- ----- ----- ----- ----- ----- ||||

Profile E

1 2 3 4 5 6 7 8 9

2 -----

3 ----- -----

4 |||| ----- ||||

5 ----- ----- ----- ||||

6 ----- ----- ----- ----- -----

7 ----- ----- ----- ----- ----- -----

8 ----- ----- ----- |||| ----- ----- -----

9 ----- |||| ----- |||| ----- ----- ----- -----

10 ----- ----- ----- ----- ----- ----- ----- ----- ||||

#################################################################

Fear during consultation

1 2 3 4 5 6 7 8 9

2 ||||

3 |||| -----

4 |||| ----- -----

5 |||| ----- ----- -----

6 |||| ----- ----- ----- -----

7 |||| ----- ----- ----- ----- -----

8 |||| ----- |||| ----- |||| ----- ||||

9 |||| ----- ----- ----- ----- ----- ----- ||||

10 |||| ----- ----- ----- ----- ----- ----- ----- -----

#################################################################

Profile A

1 2 3 4 5 6 7 8 9

2 -----

3 ----- -----

4 |||| ----- -----

5 ----- ----- ----- -----

6 ----- ----- ----- ----- -----

7 ----- ----- ----- ----- ----- -----

8 |||| ----- ----- ----- ----- ----- -----

9 ----- ----- ----- ----- ----- ----- ----- -----

10 |||| ----- ----- ----- ----- ----- ----- ----- -----

Profile B

1 2 3 4 5 6 7 8 9

2 -----

3 ----- -----

4 ----- ----- -----

5 ----- ----- ----- -----

6 |||| ----- ----- ----- -----

7 ----- ----- ----- ----- ----- -----

8 ----- ----- ----- ----- ----- ----- -----

9 ----- ----- ----- ----- ----- ----- ----- -----

10 ----- ----- ----- ----- ----- ----- ----- ----- -----

Profile C

1 2 3 4 5 6 7 8 9

2 -----

3 ----- -----

4 ----- ----- -----

5 |||| |||| ----- -----

6 ----- ----- ----- ----- -----

7 ----- ----- ----- ----- ----- -----

8 ----- |||| ----- ----- ----- ----- -----

9 ----- ----- ----- ----- ----- ----- ----- -----

10 |||| |||| ----- ----- ----- ----- ----- ----- -----

Profile D

1 2 3 4 5 6 7 8 9

2 -----

3 ----- -----

4 |||| ----- -----

5 ----- ----- ----- -----

6 ----- ----- ----- ----- -----

7 ----- ----- ----- ----- ----- -----

8 |||| ----- ----- ----- ----- |||| -----

9 ----- ----- ----- ----- ----- ----- ----- -----

10 ----- ----- ----- ----- ----- ----- ----- ----- -----

Profile E

1 2 3 4 5 6 7 8 9

2 ||||

3 ----- -----

4 |||| |||| ||||

5 ----- ----- ----- ||||

6 ----- ----- ----- |||| -----

7 ----- ----- ----- ----- ----- -----

8 |||| ----- ----- |||| ----- ----- -----

9 ----- ----- ----- |||| ----- ----- ----- -----

10 |||| ----- ----- |||| ----- ----- ----- ----- -----

#################################################################

Fear during assault

1 2 3 4 5 6 7 8 9

2 ||||

3 ----- -----

4 ----- |||| ||||

5 |||| ----- ----- ||||

6 ----- ----- ----- |||| -----

7 ----- ----- ----- ----- |||| -----

8 |||| ----- |||| |||| ----- ----- ||||

9 ----- ----- ----- |||| ----- ----- ----- -----

10 ----- ----- ----- |||| ----- ----- ----- |||| -----

#################################################################

Profile A

1 2 3 4 5 6 7 8 9

2 -----

3 ----- -----

4 ----- ----- -----

5 ----- ----- ----- -----

6 ----- ----- ----- ----- -----

7 ----- ----- ----- ----- ----- -----

8 ----- ----- ----- ----- ----- ----- ||||

9 ----- ----- ----- ----- ----- ----- ----- -----

10 ----- ----- ----- ----- ----- ----- ----- ----- -----

Profile B

1 2 3 4 5 6 7 8 9

2 ||||

3 ----- -----

4 ----- |||| -----

5 |||| ----- |||| ||||

6 ----- ----- ----- ----- -----

7 ----- ----- ----- ----- ----- -----

8 ----- ----- ----- ----- ----- ----- -----

9 |||| ----- ----- ----- ----- ----- ----- -----

10 ----- ----- ----- ----- ----- ----- ----- ----- -----

Profile C

1 2 3 4 5 6 7 8 9

2 -----

3 ----- -----

4 ----- ----- ||||

5 |||| |||| ----- ||||

6 ----- ----- ----- |||| -----

7 ----- ----- ----- ----- |||| -----

8 ----- |||| ----- |||| ----- ----- -----

9 ----- ----- ----- |||| ----- ----- ----- -----

10 ----- ----- ----- |||| ----- ----- ----- ----- -----

Profile D

1 2 3 4 5 6 7 8 9

2 -----

3 ----- -----

4 ----- ----- -----

5 ----- ----- ----- -----

6 ----- ----- ----- ----- -----

7 ----- ----- ----- ----- ----- -----

8 ----- ----- ----- |||| ----- ----- -----

9 ----- ----- ----- ----- ----- ----- ----- -----

10 ----- ----- ----- ----- ----- ----- ----- ----- -----

Profile E

1 2 3 4 5 6 7 8 9

2 -----

3 ----- -----

4 ----- ----- -----

5 ----- ----- ----- -----

6 ----- ----- ----- ----- -----

7 ----- ----- ----- ----- ----- -----

8 ----- ----- ----- ----- ----- ----- -----

9 ----- ----- ----- ----- ----- ----- ----- -----

10 ----- ----- ----- ----- ----- ----- ----- ----- -----

#################################################################

Perception of a life threat during assault

1 2 3 4 5 6 7 8 9

2 ||||

3 ----- ||||

4 ----- |||| -----

5 |||| ----- |||| ||||

6 ----- |||| ----- |||| ||||

7 ----- |||| ----- |||| |||| -----

8 |||| ----- |||| |||| ----- |||| ||||

9 |||| ----- |||| |||| |||| ----- ----- -----

10 ----- |||| ----- ----- |||| ----- ----- |||| ||||

#################################################################

Profile A

1 2 3 4 5 6 7 8 9

2 -----

3 ----- -----

4 ----- ----- -----

5 ----- ----- ----- -----

6 ----- ----- ----- ----- -----

7 ----- ----- ----- ----- ----- -----

8 |||| ----- |||| |||| ----- ----- -----

9 ----- ----- ----- ----- ----- ----- ----- -----

10 ----- ----- ----- ----- ----- ----- ----- |||| -----

Profile B

1 2 3 4 5 6 7 8 9

2 -----

3 ----- -----

4 ----- |||| -----

5 |||| ----- |||| ||||

6 ----- ----- ----- ----- ||||

7 ----- ----- ----- ----- |||| -----

8 |||| ----- |||| |||| |||| ----- -----

9 ----- ----- ----- |||| ----- ----- ----- -----

10 ----- ----- ----- ----- |||| ----- ----- |||| -----

Profile C

1 2 3 4 5 6 7 8 9

2 -----

3 ----- -----

4 ----- ----- ||||

5 |||| |||| |||| ||||

6 ----- ----- ----- |||| ||||

7 ----- ----- ----- |||| |||| -----

8 ----- ----- ----- |||| |||| ----- -----

9 ----- ----- ----- |||| |||| ----- ----- -----

10 ----- ----- ----- |||| |||| ----- ----- ----- -----

Profile D

1 2 3 4 5 6 7 8 9

2 -----

3 ----- -----

4 ----- |||| -----

5 |||| ----- |||| ||||

6 ----- ----- ----- ----- ||||

7 ----- ----- ----- ----- ----- -----

8 |||| ----- |||| |||| ----- ----- -----

9 ----- ----- ----- ----- ----- ----- ----- -----

10 ----- ----- ----- ----- |||| ----- ----- |||| -----

Profile E

1 2 3 4 5 6 7 8 9

2 ||||

3 ----- -----

4 ----- ----- -----

5 ----- ----- ----- -----

6 ----- |||| ----- ----- -----

7 ----- ----- ----- ----- ----- -----

8 |||| ----- ----- ----- ----- |||| -----

9 ----- ----- ----- ----- ----- ----- ----- -----

10 ----- ----- ----- ----- ----- ----- ----- ----- -----

#################################################################

Perception of a life threat during consultation

1 2 3 4 5 6 7 8 9

2 ||||

3 |||| ||||

4 |||| |||| -----

5 |||| ----- |||| ||||

6 |||| |||| ----- ----- ||||

7 |||| ----- ----- ----- |||| -----

8 |||| ----- |||| |||| ----- |||| ||||

9 |||| ----- ----- ----- |||| ----- ----- ||||

10 ----- |||| |||| ----- |||| ----- |||| |||| ||||

#################################################################

Profile A

1 2 3 4 5 6 7 8 9

2 ||||

3 ----- -----

4 ----- ----- -----

5 |||| ----- ----- -----

6 ----- ----- ----- ----- -----

7 ----- ----- ----- ----- ----- -----

8 |||| ----- |||| |||| ----- ----- -----

9 ----- ----- ----- ----- ----- ----- ----- ||||

10 ----- |||| ----- ----- |||| ----- |||| |||| -----

Profile B

1 2 3 4 5 6 7 8 9

2 ||||

3 ----- -----

4 ----- ----- -----

5 |||| ----- ----- ||||

6 ----- ----- ----- ----- -----

7 |||| ----- ----- ----- ----- -----

8 |||| ----- ----- |||| ----- ----- -----

9 |||| ----- ----- ----- ----- ----- ----- -----

10 ----- |||| ----- ----- |||| ----- ----- |||| ||||

Profile C

1 2 3 4 5 6 7 8 9

2 -----

3 ----- -----

4 ----- ----- -----

5 |||| |||| |||| ||||

6 ----- ----- ----- ----- ||||

7 ----- ----- ----- |||| |||| -----

8 ----- ----- ----- |||| |||| ----- -----

9 ----- ----- ----- ----- |||| ----- ----- -----

10 ----- ----- ----- ----- |||| ----- ----- ----- -----

Profile D

1 2 3 4 5 6 7 8 9

2 -----

3 ----- -----

4 ----- ----- -----

5 |||| ----- |||| ||||

6 ----- ----- ----- ----- ||||

7 ----- ----- ----- ----- ----- -----

8 |||| |||| |||| |||| ----- |||| -----

9 ----- ----- ----- ----- ----- ----- ----- ||||

10 ----- ----- ----- ----- |||| ----- ----- |||| -----

Profile E

1 2 3 4 5 6 7 8 9

2 ||||

3 |||| -----

4 |||| ----- -----

5 |||| ----- ----- -----

6 ----- |||| ----- |||| -----

7 |||| ----- ----- ----- ----- -----

8 |||| ----- ----- ----- ----- |||| -----

9 |||| ----- ----- ----- ----- ----- ----- -----

10 ----- |||| ----- |||| ----- ----- ----- |||| -----

#################################################################

functional impairment

1 2 3 4 5 6 7 8 9

2 ||||

3 |||| ||||

4 ----- |||| ||||

5 ----- |||| |||| ||||

6 |||| |||| |||| |||| ||||

7 ----- |||| |||| ----- |||| ||||

8 |||| |||| |||| |||| |||| ----- ||||

9 |||| |||| ----- |||| |||| |||| |||| ||||

10 ----- |||| |||| |||| ----- |||| |||| |||| ||||

#################################################################

Profile A

1 2 3 4 5 6 7 8 9

2 ||||

3 ----- -----

4 ----- |||| ||||

5 ----- |||| |||| -----

6 |||| ----- |||| |||| ||||

7 ----- |||| ----- ----- ----- ||||

8 |||| ----- |||| |||| |||| ----- ||||

9 ----- ----- ----- |||| |||| |||| |||| ||||

10 ----- |||| ----- ----- ----- |||| ----- |||| -----

Profile B

1 2 3 4 5 6 7 8 9

2 ||||

3 |||| -----

4 ----- |||| ||||

5 ----- |||| ----- -----

6 |||| ----- ----- |||| ||||

7 ----- |||| |||| ----- ----- ||||

8 |||| ----- |||| |||| |||| ----- ||||

9 |||| ----- ----- |||| |||| ----- |||| ||||

10 ----- |||| ----- ----- ----- |||| |||| |||| -----

Profile C

1 2 3 4 5 6 7 8 9

2 -----

3 ----- -----

4 |||| |||| ||||

5 ----- ----- |||| -----

6 ----- ----- ----- |||| -----

7 |||| |||| |||| ----- ----- ||||

8 |||| |||| ----- |||| |||| |||| ||||

9 ----- ----- ----- |||| ----- ----- |||| ||||

10 ----- ----- ----- |||| ----- ----- |||| |||| -----

Profile D

1 2 3 4 5 6 7 8 9

2 ||||

3 ----- -----

4 ----- |||| ||||

5 ----- |||| ----- -----

6 |||| ----- ----- |||| ||||

7 ----- |||| |||| ----- ----- ||||

8 |||| ----- |||| |||| |||| ----- ||||

9 ----- ----- ----- |||| ----- ----- |||| ||||

10 ----- |||| ----- |||| ----- |||| ----- |||| -----

Profile E

1 2 3 4 5 6 7 8 9

2 ||||

3 ----- ||||

4 ----- |||| ||||

5 ----- |||| ----- -----

6 |||| |||| |||| |||| ||||

7 ----- |||| |||| ----- ----- ||||

8 |||| |||| |||| |||| |||| ----- ||||

9 |||| ----- ----- |||| |||| |||| |||| ||||

10 ----- |||| ----- |||| ----- |||| ----- |||| ||||

#################################################################

psychosomatic index

1 2 3 4 5 6 7 8 9

2 ||||

3 |||| ||||

4 |||| |||| ||||

5 |||| ----- |||| -----

6 |||| ----- |||| |||| -----

7 |||| |||| |||| |||| |||| ||||

8 |||| ----- |||| |||| ----- ----- ||||

9 |||| ----- |||| ----- ----- ----- |||| -----

10 ----- ----- |||| ----- ----- |||| |||| |||| -----

#################################################################

Profile A

1 2 3 4 5 6 7 8 9

2 -----

3 |||| -----

4 ----- ----- -----

5 ----- ----- |||| -----

6 ----- ----- ----- ----- -----

7 |||| |||| |||| |||| |||| ||||

8 |||| ----- ----- ----- ----- ----- ||||

9 ----- ----- ----- ----- ----- ----- |||| -----

10 |||| ----- ----- ----- |||| ----- |||| ----- -----

Profile B

1 2 3 4 5 6 7 8 9

2 -----

3 |||| -----

4 ----- ----- ||||

5 |||| ----- ----- -----

6 |||| ----- ----- ----- -----

7 |||| |||| |||| |||| |||| ||||

8 ----- ----- ----- ----- ----- ----- ||||

9 ----- ----- ----- ----- ----- ----- |||| -----

10 ----- ----- |||| ----- |||| |||| |||| ----- -----

Profile C

1 2 3 4 5 6 7 8 9

2 -----

3 |||| ||||

4 |||| ----- ||||

5 |||| |||| |||| -----

6 ----- ----- |||| ----- -----

7 |||| |||| |||| |||| |||| ||||

8 ----- ----- |||| |||| |||| ----- ||||

9 |||| ----- |||| ----- ----- ----- |||| -----

10 ----- |||| |||| |||| |||| |||| ----- ----- ||||

Profile D

1 2 3 4 5 6 7 8 9

2 -----

3 |||| ||||

4 ----- ----- ||||

5 ----- ----- |||| -----

6 |||| ----- |||| ----- -----

7 |||| |||| |||| |||| |||| ||||

8 ----- ----- |||| ----- ----- ----- ||||

9 ----- ----- |||| ----- ----- ----- |||| -----

10 ----- ----- |||| ----- ----- ----- |||| ----- -----

Profile E

1 2 3 4 5 6 7 8 9

2 ||||

3 |||| -----

4 ----- |||| ||||

5 ----- ----- |||| -----

6 |||| ----- ----- |||| ||||

7 |||| |||| |||| |||| |||| ||||

8 |||| ----- ----- |||| |||| ----- ||||

9 ----- ----- |||| ----- ----- |||| |||| ||||

10 |||| ----- |||| ----- ----- ----- |||| |||| -----

#################################################################

quality of interaction

1 2 3 4 5 6 7 8 9

2 ||||

3 |||| ||||

4 |||| ----- ||||

5 |||| |||| |||| ||||

6 |||| |||| ----- |||| ||||

7 |||| |||| |||| |||| |||| ||||

8 |||| |||| ----- |||| |||| ----- ||||

9 ----- |||| |||| |||| |||| |||| |||| ||||

10 |||| |||| |||| |||| |||| |||| |||| |||| ||||

#################################################################

Profile A

1 2 3 4 5 6 7 8 9

2 ||||

3 ----- ||||

4 |||| ----- ||||

5 |||| ----- |||| -----

6 ----- |||| ----- |||| ||||

7 |||| |||| ----- |||| |||| -----

8 ----- |||| ----- |||| |||| ----- -----

9 ----- |||| ----- |||| |||| ----- |||| -----

10 |||| ----- |||| |||| ----- |||| |||| |||| ||||

Profile B

1 2 3 4 5 6 7 8 9

2 -----

3 ----- ||||

4 |||| ----- ||||

5 |||| |||| |||| ||||

6 ----- |||| ----- |||| ||||

7 ----- |||| ----- |||| |||| -----

8 ----- |||| ----- |||| |||| ----- -----

9 ----- ----- ----- |||| |||| ----- ----- -----

10 |||| |||| |||| |||| ----- |||| |||| |||| ||||

Profile C

1 2 3 4 5 6 7 8 9

2 -----

3 ----- ||||

4 |||| ----- ||||

5 |||| ----- |||| -----

6 ----- |||| ----- |||| ||||

7 ----- |||| ----- |||| |||| -----

8 ----- |||| ----- |||| |||| ----- -----

9 ----- ----- ----- |||| |||| ----- ----- -----

10 |||| |||| |||| |||| |||| |||| |||| |||| ||||

Profile D

1 2 3 4 5 6 7 8 9

2 ||||

3 |||| ||||

4 |||| ----- ||||

5 |||| |||| |||| ||||

6 ----- |||| ----- |||| ||||

7 |||| |||| ----- |||| |||| -----

8 |||| |||| ----- |||| |||| ----- -----

9 ----- |||| ----- |||| |||| ----- |||| -----

10 |||| ----- |||| |||| ----- |||| |||| |||| ||||

Profile E

1 2 3 4 5 6 7 8 9

2 ||||

3 ----- ||||

4 |||| ----- ||||

5 |||| |||| |||| -----

6 ----- |||| ----- |||| ||||

7 ----- |||| ----- |||| |||| -----

8 ----- |||| ----- |||| |||| ----- ||||

9 ----- |||| ----- |||| |||| ----- |||| -----

10 |||| |||| |||| |||| ----- |||| |||| |||| ||||

#################################################################

Controlled observational study of 4180 assaults’ victims who attended a medical examination during the year 2015 in a French departement of forensic medicine (Bondy, Greater Paris area). Data drawn from electronical health records.

12 Inter-rater reproducibility

**Table S4. Pairwise Kendall’s W coefficient of with 5 clusters**

|  | 2 | 3 | 4 | 5 | 6 | 7 | 8 | 9 | 10 |
| --- | --- | --- | --- | --- | --- | --- | --- | --- | --- |
| Pain during assault | | | | | | | | | |
| 1 | 0.49 | 0.47 | 0.51 | 0.51 | 0.49 | 0.45 | 0.46 | 0.55 | 0.52 |
| 2 |  | 0.45 | 0.48 | 0.5 | 0.51 | 0.48 | 0.52 | 0.56 | 0.49 |
| 3 |  |  | 0.52 | 0.52 | 0.46 | 0.54 | 0.53 | 0.51 | 0.54 |
| 4 |  |  |  | 0.52 | 0.55 | 0.54 | 0.51 | 0.54 | 0.49 |
| 5 |  |  |  |  | 0.54 | 0.5 | 0.5 | 0.52 | 0.51 |
| 6 |  |  |  |  |  | 0.47 | 0.53 | 0.51 | 0.52 |
| 7 |  |  |  |  |  |  | 0.47 | 0.43 | 0.52 |
| 8 |  |  |  |  |  |  |  | 0.48 | 0.51 |
| 9 |  |  |  |  |  |  |  |  | 0.56 |
| Pain during consultation | | | | | | | | | |
| 1 | 0.48 | 0.55 | 0.53 | 0.48 | 0.58 | 0.6 | 0.53 | 0.52 | 0.46 |
| 2 |  | 0.51 | 0.54 | 0.51 | 0.48 | 0.52 | 0.52 | 0.45 | 0.52 |
| 3 |  |  | 0.52 | 0.49 | 0.56 | 0.5 | 0.53 | 0.53 | 0.55 |
| 4 |  |  |  | 0.56 | 0.5 | 0.5 | 0.53 | 0.48 | 0.5 |
| 5 |  |  |  |  | 0.52 | 0.52 | 0.52 | 0.52 | 0.51 |
| 6 |  |  |  |  |  | 0.52 | 0.49 | 0.52 | 0.48 |
| 7 |  |  |  |  |  |  | 0.51 | 0.5 | 0.52 |
| 8 |  |  |  |  |  |  |  | 0.51 | 0.53 |
| 9 |  |  |  |  |  |  |  |  | 0.53 |
| Fear during assault | | | | | | | | | |
| 1 | 0.56 | 0.53 | 0.59 | 0.6 | 0.47 | 0.5 | 0.54 | 0.51 | 0.5 |
| 2 |  | 0.51 | 0.56 | 0.5 | 0.54 | 0.58 | 0.56 | 0.51 | 0.54 |
| 3 |  |  | 0.6 | 0.48 | 0.52 | 0.51 | 0.52 | 0.55 | 0.51 |
| 4 |  |  |  | 0.55 | 0.56 | 0.52 | 0.56 | 0.52 | 0.54 |
| 5 |  |  |  |  | 0.5 | 0.54 | 0.54 | 0.52 | 0.57 |
| 6 |  |  |  |  |  | 0.55 | 0.51 | 0.51 | 0.51 |
| 7 |  |  |  |  |  |  | 0.49 | 0.52 | 0.49 |
| 8 |  |  |  |  |  |  |  | 0.6 | 0.56 |
| 9 |  |  |  |  |  |  |  |  | 0.51 |
| Fear during consultation | | | | | | | | | |
| 1 | 0.51 | 0.5 | 0.58 | 0.54 | 0.54 | 0.56 | 0.54 | 0.5 | 0.58 |
| 2 |  | 0.53 | 0.56 | 0.57 | 0.55 | 0.57 | 0.6 | 0.55 | 0.55 |
| 3 |  |  | 0.56 | 0.54 | 0.54 | 0.56 | 0.54 | 0.55 | 0.57 |
| 4 |  |  |  | 0.56 | 0.57 | 0.52 | 0.57 | 0.56 | 0.55 |
| 5 |  |  |  |  | 0.48 | 0.5 | 0.51 | 0.51 | 0.57 |
| 6 |  |  |  |  |  | 0.56 | 0.54 | 0.51 | 0.51 |
| 7 |  |  |  |  |  |  | 0.58 | 0.57 | 0.52 |
| 8 |  |  |  |  |  |  |  | 0.55 | 0.55 |
| 9 |  |  |  |  |  |  |  |  | 0.5 |
| Perception of a life threat during assault | | | | | | | | | |
| 1 | 0.49 | 0.56 | 0.46 | 0.49 | 0.48 | 0.49 | 0.49 | 0.53 | 0.44 |
| 2 |  | 0.54 | 0.55 | 0.5 | 0.47 | 0.49 | 0.54 | 0.51 | 0.49 |
| 3 |  |  | 0.53 | 0.53 | 0.51 | 0.54 | 0.52 | 0.49 | 0.52 |
| 4 |  |  |  | 0.54 | 0.51 | 0.53 | 0.56 | 0.55 | 0.54 |
| 5 |  |  |  |  | 0.53 | 0.52 | 0.5 | 0.51 | 0.5 |
| 6 |  |  |  |  |  | 0.48 | 0.51 | 0.48 | 0.48 |
| 7 |  |  |  |  |  |  | 0.56 | 0.49 | 0.49 |
| 8 |  |  |  |  |  |  |  | 0.52 | 0.49 |
| 9 |  |  |  |  |  |  |  |  | 0.5 |
| Perception of a life threat during consultation | | | | | | | | | |
| 1 | 0.54 | 0.53 | 0.61 | 0.55 | 0.51 | 0.53 | 0.53 | 0.56 | 0.51 |
| 2 |  | 0.53 | 0.55 | 0.51 | 0.56 | 0.59 | 0.57 | 0.52 | 0.55 |
| 3 |  |  | 0.57 | 0.51 | 0.55 | 0.56 | 0.52 | 0.52 | 0.47 |
| 4 |  |  |  | 0.52 | 0.52 | 0.53 | 0.59 | 0.54 | 0.54 |
| 5 |  |  |  |  | 0.5 | 0.5 | 0.52 | 0.57 | 0.53 |
| 6 |  |  |  |  |  | 0.49 | 0.55 | 0.5 | 0.53 |
| 7 |  |  |  |  |  |  | 0.5 | 0.62 | 0.51 |
| 8 |  |  |  |  |  |  |  | 0.53 | 0.53 |
| 9 |  |  |  |  |  |  |  |  | 0.52 |
| Functional impairment | | | | | | | | | |
| 1 | 0.53 | 0.47 | 0.52 | 0.52 | 0.5 | 0.54 | 0.54 | 0.48 | 0.45 |
| 2 |  | 0.51 | 0.54 | 0.54 | 0.53 | 0.5 | 0.58 | 0.55 | 0.52 |
| 3 |  |  | 0.58 | 0.56 | 0.54 | 0.52 | 0.5 | 0.48 | 0.56 |
| 4 |  |  |  | 0.49 | 0.52 | 0.55 | 0.53 | 0.48 | 0.5 |
| 5 |  |  |  |  | 0.5 | 0.54 | 0.54 | 0.58 | 0.5 |
| 6 |  |  |  |  |  | 0.48 | 0.55 | 0.52 | 0.52 |
| 7 |  |  |  |  |  |  | 0.51 | 0.47 | 0.5 |
| 8 |  |  |  |  |  |  |  | 0.59 | 0.47 |
| 9 |  |  |  |  |  |  |  |  | 0.55 |
| Psychosomatic index | | | | | | | | | |
| 1 | 0.57 | 0.56 | 0.57 | 0.62 | 0.49 | 0.51 | 0.59 | 0.56 | 0.56 |
| 2 |  | 0.55 | 0.57 | 0.53 | 0.51 | 0.6 | 0.63 | 0.54 | 0.62 |
| 3 |  |  | 0.54 | 0.51 | 0.59 | 0.53 | 0.65 | 0.54 | 0.6 |
| 4 |  |  |  | 0.58 | 0.6 | 0.6 | 0.6 | 0.59 | 0.61 |
| 5 |  |  |  |  | 0.54 | 0.57 | 0.56 | 0.48 | 0.55 |
| 6 |  |  |  |  |  | 0.55 | 0.66 | 0.53 | 0.63 |
| 7 |  |  |  |  |  |  | 0.6 | 0.56 | 0.63 |
| 8 |  |  |  |  |  |  |  | 0.6 | 0.64 |
| 9 |  |  |  |  |  |  |  |  | 0.53 |
| Quality of interaction | | | | | | | | | |
| 1 | 0.52 | 0.46 | 0.5 | 0.56 | 0.53 | 0.51 | 0.51 | 0.5 | 0.48 |
| 2 |  | 0.47 | 0.52 | 0.49 | 0.48 | 0.48 | 0.5 | 0.52 | 0.51 |
| 3 |  |  | 0.48 | 0.49 | 0.49 | 0.51 | 0.46 | 0.54 | 0.56 |
| 4 |  |  |  | 0.54 | 0.47 | 0.5 | 0.51 | 0.48 | 0.5 |
| 5 |  |  |  |  | 0.52 | 0.48 | 0.49 | 0.54 | 0.5 |
| 6 |  |  |  |  |  | 0.57 | 0.55 | 0.44 | 0.48 |
| 7 |  |  |  |  |  |  | 0.47 | 0.54 | 0.51 |
| 8 |  |  |  |  |  |  |  | 0.5 | 0.46 |
| 9 |  |  |  |  |  |  |  |  | 0.54 |

Controlled observational study of 4180 assaults’ victims who attended a medical examination during the year 2015 in a French department of forensic medicine (Bondy, Greater Paris area). Data drawn from electronical health records.

Physicians identities are coded with integers from 1 to 10.

We assessed for pain during assault inter-rater reproducibility in the same profiles of situations of violence (SoV). Kendall’s W coefficient of concordance was computed for all pair of physicians on patients matched when they had the profile. Kendall’s W range between 0 and 1, with 1 meaning perfect agreement between physicians. Upper table shows results with 5 profiles of SoV. For example paired physicians n°2 and 3 had a Kendall coefficient equal to 0.45 with 5 profiles.

Kendall’s W coefficient of concordance was computed pairwise for all physicians to assess inter-rater concordance in similar situations. Patients were matched if they belonged to the same cluster.

**Table S5. Pairwise Kendall’s W coefficient of with 800 clusters**

|  | 2 | 3 | 4 | 5 | 6 | 7 | 8 | 9 | 10 |
| --- | --- | --- | --- | --- | --- | --- | --- | --- | --- |
| Pain during assault | | | | | | | | | |
| 1 | 0.57 | 0.52 | 0.57 | 0.52 | 0.55 | 0.55 | 0.6 | 0.56 | 0.46 |
| 2 |  | 0.5 | 0.51 | 0.53 | 0.55 | 0.6 | 0.54 | 0.54 | 0.46 |
| 3 |  |  | 0.53 | 0.56 | 0.59 | 0.51 | 0.56 | 0.57 | 0.5 |
| 4 |  |  |  | 0.57 | 0.59 | 0.56 | 0.58 | 0.54 | 0.52 |
| 5 |  |  |  |  | 0.61 | 0.52 | 0.53 | 0.56 | 0.53 |
| 6 |  |  |  |  |  | 0.53 | 0.56 | 0.54 | 0.49 |
| 7 |  |  |  |  |  |  | 0.57 | 0.59 | 0.52 |
| 8 |  |  |  |  |  |  |  | 0.5 | 0.54 |
| 9 |  |  |  |  |  |  |  |  | 0.51 |
| Pain during consultation | | | | | | | | | |
| 1 | 0.48 | 0.46 | 0.49 | 0.48 | 0.53 | 0.54 | 0.57 | 0.61 | 0.53 |
| 2 |  | 0.54 | 0.56 | 0.53 | 0.51 | 0.58 | 0.56 | 0.57 | 0.55 |
| 3 |  |  | 0.57 | 0.55 | 0.57 | 0.49 | 0.56 | 0.51 | 0.56 |
| 4 |  |  |  | 0.55 | 0.65 | 0.6 | 0.55 | 0.52 | 0.46 |
| 5 |  |  |  |  | 0.55 | 0.58 | 0.54 | 0.59 | 0.6 |
| 6 |  |  |  |  |  | 0.64 | 0.56 | 0.56 | 0.58 |
| 7 |  |  |  |  |  |  | 0.54 | 0.42 | 0.52 |
| 8 |  |  |  |  |  |  |  | 0.54 | 0.55 |
| 9 |  |  |  |  |  |  |  |  | 0.56 |
| Fear during assault | | | | | | | | | |
| 1 | 0.7 | 0.61 | 0.67 | 0.56 | 0.63 | 0.63 | 0.59 | 0.63 | 0.67 |
| 2 |  | 0.58 | 0.57 | 0.6 | 0.54 | 0.59 | 0.62 | 0.61 | 0.59 |
| 3 |  |  | 0.62 | 0.51 | 0.6 | 0.57 | 0.59 | 0.6 | 0.65 |
| 4 |  |  |  | 0.55 | 0.65 | 0.59 | 0.59 | 0.58 | 0.63 |
| 5 |  |  |  |  | 0.54 | 0.6 | 0.6 | 0.57 | 0.55 |
| 6 |  |  |  |  |  | 0.62 | 0.56 | 0.61 | 0.63 |
| 7 |  |  |  |  |  |  | 0.61 | 0.58 | 0.65 |
| 8 |  |  |  |  |  |  |  | 0.6 | 0.52 |
| 9 |  |  |  |  |  |  |  |  | 0.57 |
| Fear during consultation | | | | | | | | | |
| 1 | 0.67 | 0.55 | 0.6 | 0.62 | 0.63 | 0.54 | 0.67 | 0.49 | 0.68 |
| 2 |  | 0.55 | 0.57 | 0.66 | 0.6 | 0.63 | 0.66 | 0.63 | 0.65 |
| 3 |  |  | 0.62 | 0.47 | 0.59 | 0.57 | 0.59 | 0.63 | 0.57 |
| 4 |  |  |  | 0.61 | 0.59 | 0.63 | 0.59 | 0.65 | 0.59 |
| 5 |  |  |  |  | 0.64 | 0.62 | 0.65 | 0.61 | 0.6 |
| 6 |  |  |  |  |  | 0.6 | 0.57 | 0.58 | 0.63 |
| 7 |  |  |  |  |  |  | 0.61 | 0.62 | 0.63 |
| 8 |  |  |  |  |  |  |  | 0.55 | 0.6 |
| 9 |  |  |  |  |  |  |  |  | 0.55 |
| Perception of a life threat during assault | | | | | | | | | |
| 1 | 0.58 | 0.52 | 0.57 | 0.58 | 0.49 | 0.47 | 0.54 | 0.53 | 0.53 |
| 2 |  | 0.58 | 0.59 | 0.58 | 0.57 | 0.57 | 0.57 | 0.51 | 0.49 |
| 3 |  |  | 0.56 | 0.53 | 0.6 | 0.55 | 0.57 | 0.57 | 0.57 |
| 4 |  |  |  | 0.58 | 0.57 | 0.62 | 0.54 | 0.58 | 0.5 |
| 5 |  |  |  |  | 0.53 | 0.63 | 0.62 | 0.57 | 0.58 |
| 6 |  |  |  |  |  | 0.58 | 0.57 | 0.57 | 0.7 |
| 7 |  |  |  |  |  |  | 0.55 | 0.59 | 0.49 |
| 8 |  |  |  |  |  |  |  | 0.61 | 0.53 |
| 9 |  |  |  |  |  |  |  |  | 0.6 |
| Perception of a life threat during consultation | | | | | | | | | |
| 1 | 0.51 | 0.49 | 0.54 | 0.48 | 0.56 | 0.47 | 0.57 | 0.57 | 0.56 |
| 2 |  | 0.57 | 0.59 | 0.56 | 0.63 | 0.58 | 0.58 | 0.59 | 0.5 |
| 3 |  |  | 0.54 | 0.51 | 0.64 | 0.6 | 0.51 | 0.62 | 0.49 |
| 4 |  |  |  | 0.6 | 0.55 | 0.56 | 0.57 | 0.65 | 0.54 |
| 5 |  |  |  |  | 0.58 | 0.55 | 0.59 | 0.7 | 0.57 |
| 6 |  |  |  |  |  | 0.62 | 0.6 | 0.71 | 0.61 |
| 7 |  |  |  |  |  |  | 0.58 | 0.58 | 0.53 |
| 8 |  |  |  |  |  |  |  | 0.63 | 0.54 |
| 9 |  |  |  |  |  |  |  |  | 0.54 |
| Functional impairment | | | | | | | | | |
| 1 | 0.65 | 0.54 | 0.56 | 0.5 | 0.5 | 0.56 | 0.56 | 0.56 | 0.44 |
| 2 |  | 0.56 | 0.56 | 0.6 | 0.63 | 0.58 | 0.62 | 0.65 | 0.56 |
| 3 |  |  | 0.57 | 0.63 | 0.6 | 0.59 | 0.61 | 0.65 | 0.54 |
| 4 |  |  |  | 0.6 | 0.59 | 0.6 | 0.58 | 0.59 | 0.66 |
| 5 |  |  |  |  | 0.66 | 0.48 | 0.58 | 0.65 | 0.61 |
| 6 |  |  |  |  |  | 0.59 | 0.71 | 0.72 | 0.64 |
| 7 |  |  |  |  |  |  | 0.57 | 0.62 | 0.49 |
| 8 |  |  |  |  |  |  |  | 0.64 | 0.57 |
| 9 |  |  |  |  |  |  |  |  | 0.55 |
| Psychosomatic index | | | | | | | | | |
| 1 | 0.59 | 0.52 | 0.71 | 0.51 | 0.71 | 0.63 | 0.59 | 0.59 | 0.66 |
| 2 |  | 0.68 | 0.64 | 0.6 | 0.65 | 0.56 | 0.62 | 0.6 | 0.68 |
| 3 |  |  | 0.67 | 0.6 | 0.64 | 0.67 | 0.66 | 0.55 | 0.65 |
| 4 |  |  |  | 0.6 | 0.69 | 0.66 | 0.67 | 0.65 | 0.69 |
| 5 |  |  |  |  | 0.6 | 0.54 | 0.58 | 0.64 | 0.64 |
| 6 |  |  |  |  |  | 0.59 | 0.71 | 0.64 | 0.65 |
| 7 |  |  |  |  |  |  | 0.62 | 0.52 | 0.67 |
| 8 |  |  |  |  |  |  |  | 0.67 | 0.63 |
| 9 |  |  |  |  |  |  |  |  | 0.66 |
| Quality of interaction | | | | | | | | | |
| 1 | 0.54 | 0.5 | 0.47 | 0.56 | 0.47 | 0.45 | 0.52 | 0.6 | 0.47 |
| 2 |  | 0.45 | 0.48 | 0.49 | 0.46 | 0.6 | 0.49 | 0.5 | 0.47 |
| 3 |  |  | 0.48 | 0.54 | 0.5 | 0.48 | 0.49 | 0.56 | 0.54 |
| 4 |  |  |  | 0.52 | 0.56 | 0.53 | 0.49 | 0.53 | 0.51 |
| 5 |  |  |  |  | 0.55 | 0.56 | 0.65 | 0.45 | 0.45 |
| 6 |  |  |  |  |  | 0.55 | 0.51 | 0.53 | 0.44 |
| 7 |  |  |  |  |  |  | 0.48 | 0.6 | 0.49 |
| 8 |  |  |  |  |  |  |  | 0.54 | 0.43 |
| 9 |  |  |  |  |  |  |  |  | 0.58 |

Controlled observational study of 4180 assaults’ victims who attended a medical examination during the year 2015 in a French department of forensic medicine (Bondy, Greater Paris area). Data drawn from electronical health records.

Kendall’s W coefficient of concordance was computed pairwise for all physicians to assess inter-rater concordance in similar situations. Patients were matched if they belonged to the same cluster. Kendall’s W range between 0 and 1, with 1 meaning perfect agreement between physicians

**Table S6 Differences in scales ratings of subjective elements between physicians**

| Scales,  Median (IQR) | Physician | | | | | | | | | | *All*  *population* |
| --- | --- | --- | --- | --- | --- | --- | --- | --- | --- | --- | --- |
|  | **1** | **2** | **3** | **4** | **5** | **6** | **7** | **8** | **9** | **10** |  |
|  |  |  |  |  |  |  |  |  |  |  |  |
| Pain (assault) | 4[2-5] | 5[3-6] | 4[3-5] | 4[3-5] | 3[2-5] | 4[3-5] | 4[2-5] | 4[3-5] | 4[3-5] | 4[2-5] | 4[3-5] |
| Pain (consultation) | 2[1-3] | 3[2-4] | 3[1-4] | 4[2-4] | 3[2-4] | 3[1-4] | 2[1-4] | 3[2-4] | 2[1-3] | 3[2-4] | 3[2-4] |
| Fear (assault) | 4[0-6] | 5[1-6] | 5[1-6] | 4[0-6] | 5[3-6] | 5[2-6] | 4[0-6] | 6[3-6] | 5[2-6] | 5[3-6] | 5[1-6] |
| Fear (consultation) | 0[0-4] | 3[0-5] | 3[0-5] | 3[0-6] | 3[0-5] | 3[0-5] | 2[0-5] | 4[0-6] | 3[0-5] | 3[0-5] | 3[0-5] |
| Perception of a life  threat (assault) | 0[0-5] | 3[0-6] | 0[0-6] | 0[0-4] | 4[0-6] | 0[0-6] | 0[0-6] | 4[0-6] | 3[0-6] | 0[0-5] | 0[0-6] |
| Perception of a life  threat (consultation) | 0[0-0] | 0[0-5] | 0[0-3] | 0[0-3] | 1[0-5] | 0[0-3] | 0[0-4] | 2[0-5] | 0[0-4] | 0[0-0] | 0[0-4] |
| Functional impairment | 1[0-1] | 2[1-3] | 1[0-2] | 0[0-1] | 1[0-1] | 2[1-3] | 0[0-1] | 2[2-3] | 1[1-2] | 1[0-2] | 1[0-2] |
| Quality of interaction  between patient  and physician | 5[4-5] | 6[5-6] | 5[5-5] | 6[5-6] | 6[6-6] | 5[4-5] | 5[4-5] | 5[5-5] | 5[5-5] | 6[6-6] | 5[5-6] |
| Psychosomatic index | 2[0-3] | 3[1-4] | 3[3-4] | 2[2-3] | 3[2-3] | 3[1-4] | 0[0-1] | 3[1-4] | 3[1-3] | 2[0-4] | 3[1-3] |

Controlled observational study of 4180 assaults’ individuals exposed to violence who attended a medical examination during the year 2015 in a French department of forensic medicine (Bondy, Greater Paris area). Data drawn from electronical health records.

We present scale medians for each physician and in the overall population. Kruskall-wallis tests for differences in median were significant with all p-values < 0.001.

**Table S7 Intra-rater reproducibility of scales for similar situations of violence**

| Scales |  |  | |  |  | 5 profiles | | | | | | | | | |
| --- | --- | --- | --- | --- | --- | --- | --- | --- | --- | --- | --- | --- | --- | --- | --- |
|  | | | | |  | **1** | **2** | **3** | **4** | **5** | **6** | **7** | **8** | **9** | **10** |
| **Pain** | | | **assault** | | | 0.52 | 0.55 | 0.54 | 0.52 | 0.53 | 0.50 | 0.61 | 0.51 | 0.52 | 0.55 |
|  | | | **consultation** | | | 0.48 | 0.50 | 0.54 | 0.51 | 0.47 | 0.50 | 0.49 | 0.50 | 0.53 | 0.54 |
| **Fear** | | | **assault** | | | 0.58 | 0.58 | 0.59 | 0.56 | 0.48 | 0.60 | 0.57 | 0.57 | 0.52 | 0.54 |
|  | | | **consultation** | | | 0.62 | 0.61 | 0.58 | 0.59 | 0.57 | 0.51 | 0.57 | 0.56 | 0.53 | 0.54 |
| **Perception  of a life threat** | | | **assault** | | | 0.53 | 0.53 | 0.46 | 0.51 | 0.54 | 0.49 | 0.46 | 0.51 | 0.48 | 0.52 |
|  | | | **consultation** | | | 0.54 | 0.55 | 0.53 | 0.57 | 0.51 | 0.52 | 0.53 | 0.54 | 0.62 | 0.46 |
| **Functional impairment** | | |  | | | 0.52 | 0.58 | 0.51 | 0.55 | 0.56 | 0.60 | 0.53 | 0.59 | 0.55 | 0.58 |
| **Quality of interaction between patient and physician** | | |  | | | 0.52 | 0.46 | 0.56 | 0.55 | 0.62 | 0.53 | 0.50 | 0.51 | 0.53 | 0.46 |
| **Psychosomatic index** | | |  | | | 0.60 | 0.53 | 0.54 | 0.62 | 0.52 | 0.53 | 0.56 | 0.66 | 0.57 | 0.65 |
|  | | | | |  | **800 profiles** | | | | | | | | | |
|  | | | | |  | **1** | **2** | **3** | **4** | **5** | **6** | **7** | **8** | **9** | **10** |
| **Pain** | | | | | **assault** | 0.62 | 0.56 | 0.51 | 0.55 | 0.59 | 0.64 | 0.61 | 0.52 | 0.53 | 0.48 |
|  | | | | | **consultation** | 0.64 | 0.61 | 0.58 | 0.60 | 0.60 | 0.53 | 0.60 | 0.58 | 0.59 | 0.42 |
| **Fear** | | | | | **assault** | 0.50 | 0.59 | 0.50 | 0.61 | 0.61 | 0.57 | 0.66 | 0.52 | 0.56 | 0.62 |
|  | | | | | **consultation** | 0.62 | 0.63 | 0.54 | 0.69 | 0.63 | 0.66 | 0.66 | 0.57 | 0.57 | 0.59 |
| **Perception  of a life threat** | | | | | **assault** | 0.48 | 0.56 | 0.57 | 0.60 | 0.57 | 0.44 | 0.60 | 0.51 | 0.56 | 0.57 |
|  | | | | | **consultation** | 0.39 | 0.55 | 0.52 | 0.63 | 0.68 | 0.54 | 0.58 | 0.54 | 0.54 | 0.56 |
| **Functional impairment** | | | | |  | 0.66 | 0.61 | 0.64 | 0.58 | 0.64 | 0.68 | 0.64 | 0.65 | 0.62 | 0.54 |
| **Quality of interaction between patient and physician** | | | | |  | 0.53 | 0.64 | 0.49 | 0.52 | 0.50 | 0.46 | 0.51 | 0.47 | 0.59 | 0.45 |
| **Psychosomatic index** | | | | |  | 0.60 | 0.67 | 0.69 | 0.74 | 0.58 | 0.70 | 0.58 | 0.66 | 0.56 | 0.76 |

Controlled observational study of 4180 assaults’ individuals exposed to violence who attended a medical examination during the year 2015 in a French department of forensic medicine (Bondy, Greater Paris area). Data drawn from electronical health records.

Physicians identities are coded with integers from 1 to 10.

We assessed intra-rater reproducibility of scales in the same profiles of situations of violence (SoV). Kendall’s W coefficient of concordance was computed for each physician on patients matched when they had the same profile of SoV. Kendall’s W range between 0 and 1, with 1 meaning perfect agreement between physicians. Upper table shows results with 5 profiles of SoV. For example physicians n°2 had for pain during assault a Kendall coefficient equal to 0.55 with 5 profiles.
