# Supplementary material for: Functional Impairment in Individuals Exposed to Violence Based on Electronical Forensic Medical Record Mining and Their Profile Identification: Controlled Observational Study
Source: JMIR Public Health Surveill. 2024 Sep 27;10:e43563. doi: 10.2196/43563 (PMC11470214; doi:10.2196/43563)
Supplement: Multimedia Appendix 2 [file publichealth_v10i1e43563_app2.docx]

Differences in ratings of subjective elements between physicians in the same profile of situations of violence.^a,b.^

| Physician identity^c^ | | 1 | 2 | 3 | 4 | 5 | 6 | 7 | 8 | 9 |
| --- | --- | --- | --- | --- | --- | --- | --- | --- | --- | --- |
| **All population** | | | | | | | | | | |
|  | 2 | * | — | — | — | — | — | — | — | — |
|  | 3 | ## | * | — | — | — | — | — | — | — |
|  | 4 | ## | * | ## | — | — | — | — | — | — |
|  | 5 | ## | * | * | ## | — | — | — | — | — |
|  | 6 | * | ## | ## | ## | * | — | — | — | — |
|  | 7 | ## | * | ## | ## | ## | * | — | — | — |
|  | 8 | * | ## | ## | ## | * | ## | * | — | — |
|  | 9 | ## | * | ## | ## | ## | ## | ## | ## | — |
|  | 10 | ## | * | ## | ## | ## | * | ## | * | ## |
| **Profile A** | |  |  |  |  |  |  |  |  |  |
|  | 2 | ## | — | — | — | — | — | — | — | — |
|  | 3 | ## | ## | — | — | — | — | — | — | — |
|  | 4 | ## | ## | ## | — | — | — | — | — | — |
|  | 5 | ## | * | ## | ## | — | — | — | — | — |
|  | 6 | ## | ## | ## | ## | ## | — | — | — | — |
|  | 7 | ## | ## | ## | ## | ## | ## | — | — | — |
|  | 8 | ## | ## | ## | ## | ## | ## | ## | — | — |
|  | 9 | ## | ## | ## | ## | ## | ## | ## | ## | — |
|  | 10 | ## | ## | ## | ## | ## | ## | ## | ## | ## |
| **Profile B** | |  |  |  |  |  |  |  |  |  |
|  | 2 | * | — | — | — | — | — | — | — | — |
|  | 3 | ## | ## | — | — | — | — | — | — | — |
|  | 4 | ## | ## | ## | — | — | — | — | — | — |
|  | 5 | ## | ## | ## | ## | — | — | — | — | — |
|  | 6 | ## | # | # | # | # | — | — | — | — |
|  | 7 | # | # | # | # | # | # | — | — | — |
|  | 8 | # | # | # | # | # | # | # | — | — |
|  | 9 | # | # | # | # | # | # | # | # | — |
|  | 10 | # | # | # | # | # | # | # | # | # |
| **Profile C** | |  |  |  |  |  |  |  |  |  |
|  | 2 | ## | — | — | — | — | — | — | — | — |
|  | 3 | ## | ## | — | — | — | — | — | — | — |
|  | 4 | ## | ## | ## | — | — | — | — | — | — |
|  | 5 | ## | ## | ## | ## | — | — | — | — | — |
|  | 6 | # | # | # | # | # | — | — | — | — |
|  | 7 | # | # | # | # | # | # | — | — | — |
|  | 8 | * | # | # | * | * | # | * | — | — |
|  | 9 | # | # | # | # | # | # | # | # | — |
|  | 10 | # | # | # | # | # | # | # | # | # |
| **Profile D** | |  |  |  |  |  |  |  |  |  |
|  | 2 | * | — | — | — | — | — | — | — | — |
|  | 3 | # | * | — | — | — | — | — | — | — |
|  | 4 | # | * | # | — | — | — | — | — | — |
|  | 5 | # | # | # | # | — | — | — | — | — |
|  | 6 | # | # | # | # | # | — | — | — | — |
|  | 7 | # | # | # | # | # | # | — | — | — |
|  | 8 | # | # | # | # | # | # | # | — | — |
|  | 9 | # | # | # | # | # | # | # | # | — |
|  | 10 | # | * | # | # | # | # | # | # | # |
| **Profile E** | |  |  |  |  |  |  |  |  |  |
|  | # | — | — | — | — | — | — | — | — | — |
|  | # | # | — | — | — | — | — | — | — | — |
|  | # | # | # | — | — | — | — | — | — | — |
|  | # | * | # | # | — | — | — | — | — | — |
|  | # | # | # | # | # | — | — | — | — | — |
|  | # | # | # | # | # | # | — | — | — | — |
|  | # | # | # | # | * | # | # | — | — | — |
|  | # | # | # | # | # | # | # | # | — | — |
|  | # | # | # | # | # | # | # | # | # | — |

^a^Controlled observational study of 4180 individuals who experienced assaults who attended a medical examination during the year 2015 in a French department of forensic medicine (Bondy, Greater Paris area). Data were drawn from electronic health records.

^b^We assessed pairwise differences between physicians first for all situations of violence and second within each situation. * is coding for a significant association (ie, *P*<.05) and # is coding for a nonsignificant association (ie, *P≥*.05), when performing Conover post hoc analysis of differences in the median. All physicians had seen more than 30 patients in each situation of violence. Here we present results for pain during the assault; for example, paired physicians 2 and 3 had significantly (ie, *P*<.05) different ratings in the overall population, but these differences became nonsignificant within all profiles but profile D.

^c^Physicians’ identities are coded with integers from 1 to 10.
